# Forensic mental health service use in early psychosis: A scoping review

**DOI:** 10.1007/s00127-025-03038-4

**Published:** 2026-01-19

**Authors:** Rebecca Rodrigues, Jared C. Wootten, Kelly K. Anderson, Saverio Stranges, Piotr Wilk, Michael D. Freeman, Maurice P. Zeegers

**Affiliations:** 1 Department of Epidemiology, Care and Public Health Research Institute, Maastricht University, Maastricht, ON The Netherlands; 2 Department of Epidemiology and Biostatistics, Schulich School of Medicine & Dentistry, Western University, London, ON Canada; 3 Department of Psychiatry, Schulich School of Medicine & Dentistry, Western University, London, ON Canada; 4 Lawson Health Research Institute, London, Canada; 5 Department of Family Medicine, Schulich School of Medicine & Dentistry, Western University, London, ON Canada; 6 Department of Medicine, Schulich School of Medicine & Dentistry, Western University, London, ON Canada; 7 Department of Clinical Medicine and Surgery, Federico II University, Naples, Italy; 8 Department of Paediatrics, Schulich School of Medicine & Dentistry, Western University, London, ON Canada; 9 Faculty of Forensic and Legal Medicine, Royal College of Physicians, London, UK; 10 School of Nutrition and Translational Research in Metabolism, Maastricht University, Maastricht, The Netherlands

**Keywords:** Early psychosis, First-episode psychosis, Forensic mental health services, Forensic hospitalization

## Abstract

**Purpose:**

Although early psychosis and forensic mental health service use are well-studied concepts individually, less is known about their co-occurrence. We aimed to scope evidence on the overlap between early psychosis and forensic mental health service use, including evidence on forensic mental health service use within early psychosis groups and early psychosis cases within forensic mental health services. We further sought evidence specifically on prevalence or incidence, and associated risk factors and outcomes of forensic mental health service use in early psychosis.

**Methods:**

We searched MEDLINE, EMBASE, CINAHL, PsycINFO, Scopus, Web of Science, Google Scholar, and thesis databases, with forward and backward citation searching. Eligible studies identified an early psychosis group and reported on forensic mental health service use.

**Results:**

We included 74 sources, primarily European (47%) and cross-sectional (69%). Most examined homicide offenders with psychosis (62%), among whom a substantial proportion were early psychosis. Fewer studies examined broader forensic mental health service populations. Among studies on early psychosis populations (*n* = 5), two reported forensic hospitalization incidence (5%-6%), and three identified risk factors (male sex, Black-Caribbean race/ethnicity, and prior police contact). One study linked forensic hospitalization with greater inpatient service use over follow-up.

**Conclusion:**

Although substantial evidence highlights an increased risk of committing homicide in early psychosis relative to chronic psychosis, evidence is limited on forensic mental health service use in early psychosis populations. Cohort studies using a broader mental health system perspective are needed to clarify the frequency, risk factors, and associated outcomes of forensic mental health service use in early psychosis.

**Supplementary Information:**

The online version contains supplementary material available at https://doi.org/10.1007/s00127-025-03038-4.

## Introduction

Early psychosis, the 2- to 5-year period following psychosis onset, represents a “critical period” for improving long-term outcomes [1, 2]. Early psychosis intervention services have been established worldwide to capitalize on this window of opportunity, with the aims of reducing treatment delays and providing comprehensive wraparound care to facilitate recovery [3]. The advent of early intervention has led to an interest in pathways to care and service use patterns early in the course of illness. Hospitalization, emergency service use, and coercive measures such as involuntary hospitalization are common in early psychosis [4]. Evidence suggests that approximately half of people with early psychosis are hospitalized at least once [5] and 22% to 62% are admitted involuntarily [6]. Involuntary admission is associated with higher risk of experiencing seclusion or control interventions [7]. The nature and quality of care experienced in early psychosis can impact long-term trajectories [8], and coercive interventions can be particularly distressing [9, 10], potentially initiating cycles of coercion, disengagement, and relapse [11, 12].

Although hospitalization and involuntary admission in early psychosis have been widely studied, contact with the forensic mental health system (the intersection of mental health and criminal justice systems) [13–15] is often overlooked. The forensic mental health system serves those with a mental illness who committed a crime and are assessed for fitness to stand trial and/or criminal responsibility. For those ultimately found not guilty on the grounds of mental illness - a term that varies internationally (e.g., “not guilty by reason of insanity”, or “not criminally responsible” in some jurisdictions) - this often results in detention in a forensic hospital or unit [13, 15]. The aim of forensic psychiatric services is to balance treatment of people suffering from a mental disorder, along with mitigating risk to the public, leading to greater restrictions and longer confinement than in general psychiatry [16] - ranging from 1 year to 10 years on average across European countries [17]. Forensic mental health service users also face greater levels of social and structural stigma [18]. In early psychosis, contact with forensic mental health services may hinder access to early psychosis intervention services, as some programs exclude people with forensic involvement [19, 20].

Young people with early psychosis may be at high risk of contact with forensic mental health services. In Denmark, as many as one in five people have a history of offending prior to their first psychiatric contact for psychotic disorder [21]. In Canada, half of people with early psychosis have police contact on the pathway to care [22], and early psychosis is recognized as a high-risk time for violence [23], all of which may increase the likelihood of forensic mental health system involvement [15]. Within forensic inpatient settings, people with psychotic disorders account for most forensic admissions, up to 80% in Canada [15] and New Zealand [24], although it is unclear what proportion are early psychosis. Several regions, including Canada, the United States, and Western Europe, have reported increased demand for forensic mental health services in recent years [25]. Canadian data indicate that this growth can be attributed to people who are younger, abuse substances, are of diverse ethno-racial backgrounds, and have committed low-level violent offences [26] - characteristics that overlap with early psychosis populations, suggesting that this growth may in part involve early psychosis cases.

Overall, it is unclear what evidence is available on early psychosis and the forensic mental health system. Recent systematic reviews have broadly examined related topics, including psychosis and violence [27, 28], criminal offending [29], and the prevalence of violence in early psychosis [30]. However, these studies do not explore both the early phase of illness and contact with forensic mental health services, specifically. It is unclear what evidence is available regarding basic epidemiological data on early psychosis and forensic mental health service use, including frequency of contact with forensic mental health services in early psychosis, or conversely, how common early psychosis is within forensic mental health services. This underexplored aspect of the broader literature on psychosis, violence and criminal offending has specific clinical, service provision, and policy implications. Thus, there is a need to identify and synthesize evidence to inform future studies.

Our objective was to scope the available evidence on the co-occurrence of early psychosis and forensic mental health services, including evidence on forensic mental health service use within early psychosis populations, and early psychosis within forensic mental health services. A sub-aim of this study was to specifically identify evidence on prevalence or incidence of contact with forensic mental health services within early psychosis populations, and any risk factors or outcomes associated with forensic mental health service use in this group. This sub-aim was included to inform future studies on early psychosis cohorts, including pathways into forensic mental health services in this population and the implications of forensic mental health system involvement during the early phase of illness.

## Method

We used scoping review methodology to gather evidence rather than a systematic review for several reasons. First, the literature on this topic is limited and conceptually broad, spanning multiple disciplines, jurisdictions, and populations. Definitions for both early psychosis and forensic mental health services may vary considerably across studies and international contexts. Given such variation, a scoping review is well suited to map the existing evidence, clarify definitions, and identify knowledge gaps. Finally, a scoping review approach can provide a foundation to inform future systematic reviews and research in this area. We followed scoping review methodology in accordance with the Joanna Briggs Institute [31]. We developed and pre-registered a protocol for this review *a priori* (https://osf.io/vy6ez/) [32]. This review follows the reporting guidelines in the PRISMA extension for Scoping Reviews (PRISMA-ScR; online supplement Table S1) [33].

### Inclusion criteria

We followed the participant, concept, and context framework for scoping review inclusion criteria [31]. We included studies if participants included an early psychosis group, referring to the 2- to 5-year period after onset of full-threshold disorder. This is closely related to the concept of “first-episode psychosis,” similarly referring to the period of first onset. Several operational definitions exist for early or first-episode psychosis based on prior mental health service contact, antipsychotic treatment history, or duration of illness [34]. Our original inclusion criteria required participants to be within five years of psychosis onset, experiencing a first psychiatric hospitalization for psychosis, or recruited via early psychosis intervention services. However, during screening, we encountered limitations with these criteria. Duration of illness was rarely reported, and many studies reported prior mental health service contact and/or treatment history. In some cases, duration of illness conflicted with the first hospitalization criterion - for example, a first admission sample including patients with a duration of untreated psychosis of more than five years. We also identified studies of patients with psychotic disorder whose first mental health contact occurred as forensic mental health outpatients, rather than through hospitalization. We revised our inclusion criteria to address inconsistencies, to be as inclusive as possible, and to better align with the forensic literature [35]. Final inclusion criteria defined early psychosis as no prior psychiatric treatment for psychosis (including inadequate treatment for acute symptoms to stabilize), no prior contact with mental health services for psychosis, no prior psychiatric hospitalization for psychosis, or were recruited via early psychosis intervention services. We removed the duration of illness criterion to avoid conflicts with other inclusion criteria but extracted this information where available to describe sample characteristics. We included studies reporting stratified data for first- and multi-episode psychosis cases.

The concept for study inclusion was contact with the forensic mental health system, referring to specialized services that assess and treat people with mental disorders whose behaviour has led, or could lead, to offending [36]. We included studies that identified forensic mental health system patients, including patients undergoing forensic psychiatric assessment, found not guilty due to a mental illness, found unfit to stand trial, or admitted to a forensic hospital/unit. Studies stratifying hospitalizations by forensic and civil involuntary sections of mental health legislation were included. We did not include evidence from prison or correctional settings, as people referred to prison mental health services may not be subject to the same legal frameworks or treatment protocols as in specialized forensic mental health services. Since this review focused on forensic mental health service use rather than criminal offending, studies examining specific offender groups (e.g., violence, homicide) were included only if participants were ascertained through forensic mental health service contact. Offender studies without such specification were excluded. Overall, studies allowing identification of a co-occurrence between early psychosis participants and forensic mental health services were included. This included studies with stratified data such that either an early psychosis group could be identified within forensic mental health service users, or forensic mental health service users could be identified within an early psychosis group. We considered studies within any context for inclusion (for example, any geographic region, inpatient and outpatient populations).

## Search strategy

We developed a search strategy using keywords and subject headings specific to each database for the concepts “psychosis,” “early/first-episode psychosis,” and “forensic mental health services” (online supplement, Table S2). We searched MEDLINE, EMBASE, PsycINFO, CINAHL, Scopus, and Web of Science. We searched dissertation and thesis databases (ProQuest Dissertations & Theses Global) and Google Scholar (limiting to the first 200 records) for sources of unpublished studies/grey literature. The database search was conducted on March 16, 2023, and updated on October 24, 2025. We then conducted forward and backward citation searching of all included sources and relevant reviews [23, 37, 38] using Web of Science or Scopus. We did not apply any language or date restrictions.

## Source of evidence selection

All records identified through searching were uploaded to Covidence (www.covidence.org). Two reviewers (RR, JCW) independently screened titles/abstracts, in duplicate, against study eligibility criteria. Full texts were retrieved and independently screened by two reviewers for eligibility. Reasons for exclusion at the full text stage were recorded. Disagreements arising between reviewers at each selection stage were resolved through discussion between reviewers.

## Data charting

Data charting was conducted by the primary author (RR), and extracted data were verified by a second reviewer (JCW) using a piloted form in Covidence. Data extracted included study location, objectives, setting, design, timeframe, data source, and population. We extracted data on early psychosis and forensic mental health service use definitions, sample sizes, study population details (age, sex, and diagnoses), and duration of psychotic illness. We also extracted findings relevant to forensic mental health service use in early psychosis.

## Synthesis of results

Results were synthesized in narrative format and using descriptive statistics. To facilitate evidence synthesis, we grouped evidence sources according to population type, identified based on the study population and aims. Population categories included: (1) early psychosis population; (2) offence-specific population (groups committing specific offences which resulted in forensic hospitalization/not guilty verdicts on the grounds of mental illness, including homicide/severe violence, domestic homicide/filicide/parricide, violence, and sex-offences); (3) forensic mental health services population (groups defined by contact with forensic mental health services, and not defined by specific offences, such as forensic psychiatric inpatients, outpatient forensic mental health services population, and forensic psychiatric assessments); and (4) other (sources not fitting into the above categories). For studies with overlapping population categories (for example, offence-specific groups were a subset of forensic mental health service populations), the main population based on the study aims was selected. This approach reflects the population definitions in included studies, differentiating studies that focused on forensic mental health services versus studies focusing on specific offences which were ascertained through forensic mental health service contact. Given the specific sub-question of this review on the incidence/prevalence, risk factors, and outcomes for forensic mental health service use within an early psychosis population, studies focusing on an early psychosis population are reported in more detail.

## Results

**Fig. 1:**
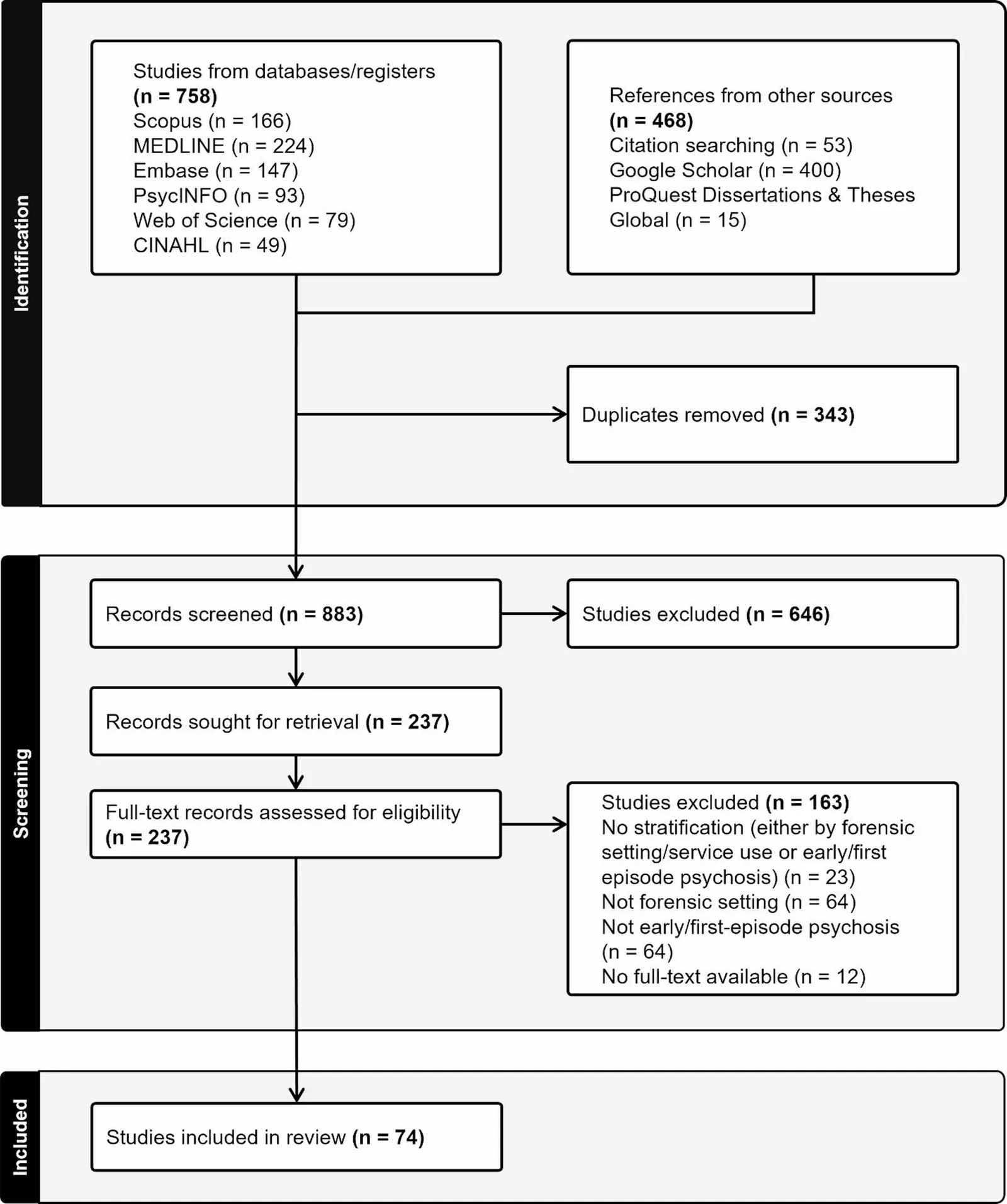
Flow diagram of study selection

We retrieved 883 unique records and excluded 646 at the title/abstract screening stage. Of 237 full texts reviewed, 74 met inclusion criteria (Fig. 1) [35, 37, 39–110]. Aggregate characteristics of included evidence are summarized in Table 1, and characteristics of each study are available in the online supplement (Table S3). Most sources were journal articles (93%), along with two books/book chapters (3%), one dissertation (1%), one column (1%), and one conference abstract (1%). Studies were primarily from Europe (47%) and cross-sectional in design (69%). Study periods spanned from pre-1950 to post-2020, with the largest proportion from 2000 to 2009 (44%). Reports/files (e.g., medico-legal reports, hospital charts) were the most common data source (79%). In 82% of studies, the early psychosis group was identified as a descriptive characteristic of the sample, whereas for 18% it was a comparison group, and for 15%, early psychosis defined the study population. Definitions of early psychosis included no prior treatment (55%), no prior hospitalization (31%), and no prior mental health service contact (27%). Forensic mental health service use was primarily defined by admission to a forensic hospital/unit (43%) or being found not guilty due to a mental illness (28%). The mean sample size was 410 (SD = 1,312). Most studies included people with schizophrenia spectrum disorders (81%), while 41% also included other non-affective psychoses and 18% included affective psychoses. Study samples had a mean age of 34 years (SD = 6.4) and were primarily male (mean 82%, SD = 27%). Mean duration of illness was 6.7 years (SD = 4.3; *n* = 12 studies), and 1.9 years in early psychosis groups (SD = 1.7, *n* = 10 studies). Findings from individual evidence sources are summarized in the online supplement (Table S4).

**Table 1:** Characteristics of included evidence sources (*N* = 74)

Characteristic		*N* (%) or mean (SD)
Publication type	Book or book chapter	2 (3%)
	Column	1 (1%)
	Conference abstract	1 (1%)
	Dissertation	1 (1%)
	Journal article	69 (93%)
Region	Africa	3 (4%)
	Asia	8 (11%)
	Australia/Oceania	10 (14%)
	Europe	35 (47%)
	International	6 (8%)
	North America	9 (12%)
	South America	3 (4%)
Study design	Cross-sectional	51 (69%)
	Case-control	4 (5%)
	Cohort	4 (5%)
	Systematic review	4 (5%)
	Meta-analysis	2 (3%)
	Case study/series	13 (18%)
	Narrative review	3 (4%)
	Pre/post	1 (1%)
	Other	1 (1%)
Study timeframe	Before 1950	1 (1%)
	1950–1959	2 (3%)
	1960–1969	7 (10%)
	1970–1979	9 (12%)
	1980–1989	18 (25%)
	1990–1999	30 (41%)
	2000–2009	32 (44%)
	2010–2019	24 (33%)
	2020 onwards	5 (7%)
	Not reported	16 (22%)
Data source	Registers/administrative databases	9 (13%)
	Reports/files	56 (79%)
	Patient survey/interview	14 (20%)
	Published literature	5 (7%)
	Not reported	5 (7%)
	Media	2 (3%)
Inclusion of early psychosis group in study	Characteristic of study sample	59 (82%)
	Comparison group	13 (18%)
	Study population definition	11 (15%)
Early psychosis definition	No previous treatment	41 (55%)
	No prior contact with mental health services	20 (27%)
	No prior psychiatric hospitalization	23 (31%)
	Other	10 (14%)
Forensic mental health service use definition	Found not guilty due to mental illness	21 (28%)
	Found unfit to stand trial	3 (4%)
	Admitted to forensic hospital/unit	32 (43%)
	Admitted under forensic section of mental health act	2 (3%)
	Forensic psychiatric assessment	30 (41%)
	Other	6 (8%)
Sample size	Mean (SD)	409.6 (1,311.7)
Psychotic disorder diagnoses included	Schizophrenia spectrum disorders	59 (81%)
	Other non-affective psychosis	30 (41%)
	Affective psychosis	13 (18%)
	Not reported	16 (22%)
Age (years)	Mean age (SD)^1^	33.9 (6.4)
Sex	Percent male, mean (SD)^2^	81.6 (26.5)
	Percent female, mean (SD)^2^	18.0 (26.5)
Duration of psychotic illness (years)	Total sample, mean (SD)^3^	6.7 (4.3)
	Early psychosis group, mean (SD)^4^	1.9 (1.7)

SD = standard deviation

^1^*n* = 50 studies

^2^*n* = 66 studies

^3^*n* = 12 studies

^4^*n* = 10 studies

## Early psychosis population

Five studies (8%) provided evidence on forensic mental health service use within early psychosis populations (Table 2). Two reported on the incidence of forensic hospitalization in early psychosis, including 4.7% of an early psychosis cohort in the United Kingdom (UK; *n* = 253) [71], and 5.6% of index admissions in an early psychosis cohort in Israel (*n* = 10,591) [68]. Incidence varied by sex, with a higher proportion of males admitted with forensic status (8.1%) versus females (1.8%) [68]. Forensic index admission was associated with more hospital days per year, longer initial stays, and for males only, more admissions per illness year relative to voluntary and involuntary index admissions over 4- to 18-year follow up [68].

**Table 2:** Evidence focusing on early psychosis populations, examining the incidence/prevalence of forensic mental health service use, associated factors, and/or outcomes

Author, year	Country	Population	Study design	Setting	Data sources	Early psychosis definition	Forensic concept definition	*N*	Findings
Bourget 2004 [47]	Canada	Cases referred for a forensic psychiatric exam charged with homicide or attempted homicide, and diagnosed with a first psychotic episode after the homicidal event	Case series	Not reported	Forensic psychiatric examination records	No prior psychiatric treatment for psychosis	Forensic psychiatric assessment	22	All cases presented with insidious illness onset, which went unrecognized until the homicidal event. Most patients (68%) were diagnosed with schizophrenia. Only 4 patients (18%) had past contacts with psychiatric services (for somatic complaints, substance abuse, and anxiety/depression). Only one patient had a history of violent behaviour.
Humphreys 1994 [62]	United Kingdom	Patients with a first episode of schizophrenia (15–70 years old) with no previous hospitalization and offending history within 5 years prior to first admission	Cross-sectional	Multi-centre within one London borough	Interviews (patients, families, and nursing staff)	No prior psychiatric treatment for psychosis; No prior hospitalization for psychosis	Admitted to forensic hospital or unit	31	15/31 patients (48%) committed offences associated with psychotic symptoms or were ill at the time of the offence. Only 6/15 patients (40%) were diverted to forensic mental health services via hospital order. In other cases, the mental disorder went unnoticed.
Levine 2008 [68]	Israel	First psychiatric hospitalization for schizophrenia spectrum disorders (1978-1992)	Cohort	Population-based	National registry of all psychiatric hospitalizations	No prior hospitalization for psychosis	Admitted to forensic hospital or unit	10,591	5.6% of patients were admitted with forensic status at first admission (8.1% of males and 1.8% of females). Over 4- to 18-year follow up: 35% of males and 16% of females with an index forensic admission had a second forensic admission. A forensic index admission was associated with a higher average number of hospital days per year and longer length of first stay relative to voluntary and involuntary index admissions. For males, a forensic index admission was associated with more admissions per illness year.
Macmillan 1987 [71]	United Kingdom	Patients (15–70 years old) hospitalized for a first psychotic illness, excluding organic psychosis (1979-1981)	Cross-sectional; Cohort	Multi-centre within one London borough	Interviews (patients, relatives, and nursing staff), case notes	No prior psychiatric treatment for psychosis	Admitted under forensic sections of the Mental Health Act	253	4.7% were hospitalized under a forensic section of the Mental Health Act. Among patients with police contact prior to admission, 22% were hospitalized under forensic section of the Mental Health Act.
McGovern 1991 [73]	United Kingdom	First psychiatric admission for schizophrenia (1980-1983)	Cohort	One hospital (Birmingham)	Case notes	No prior hospitalization for psychosis	Admitted under forensic sections of the Mental Health Act	61	On first psychiatric admission, Black-Caribbeans were more likely to be detained under a forensic section compared to the white group (10% vs. 0%) and over 4- to 8- year follow-up. 21% of Black Caribbeans and 3% of the white group were admitted under forensic sections at any point over the study period. Only males were detained in either group, with 1/3 of Black-Caribbean males having been detained (and 5% of the white group).

Regarding factors associated with forensic mental health service use, a UK study found that a Black-Caribbean early psychosis group were more likely to have a forensic hospitalization compared to a white group, both at first admission and over a 4- to 8-year follow up [73]. The detained Black-Caribbean group was entirely male, and overall, one-third of Black-Caribbean males had a forensic admission at any point during the study, compared to 5% of white males [73]. In another UK early psychosis sample with a history of offending while ill, only 40% received a hospital order (indicating involvement with forensic mental health services), highlighting that early psychosis may go unrecognized [62]. Finally, a case series of people with early psychosis who underwent forensic psychiatric assessment after committing homicide found a high proportion of schizophrenia (68%), insidious onset of illness, and minimal prior psychiatric contact (18%) [47].

## Offence-specific population

Fifty-one studies (69%) provided evidence on forensic mental health service use and early psychosis within offence-specific populations (Table 3). Most studies (*n* = 46; 62% of all included studies) focused on populations committing homicide/severe violence. Among these, 36 studies focused on any type of homicide/severe violence and 10 studies on specific subtypes such as domestic homicide, filicide, or parricide. A meta-analysis found that that 38.5% (95% CI 31.3%−46.5%) of homicide offenders with psychosis were early in the course of illness, and that homicide rates in early psychosis were 15 times higher compared to previously treated psychosis [35]. Additional studies reported wide variation in early psychosis prevalence, ranging from 12% to 70% of homicide/severe violence offenders across 21 studies [39, 41, 45, 51, 53, 58, 63, 67, 70, 75, 77, 79, 80, 82, 84, 85, 87, 94, 97, 99, 100].

**Table 3:** Summary of evidence regarding forensic mental health service use in early psychosis in offence-specific populations (*N* = 51)

Evidence population category	n (% of all included studies, *N*=74)	Studies	Summary of evidence
Homicide/severe violence	36 (49%)	Amani 2022, Azoulay 2020, Balcioglu 2024, Barbieri 2022, Belli 2010, Böker & Häfner 1982, Bourget 2004, Carabellese 2021, Chen 2018, Dolan 1996, Erb 2001, Golenkov 2011, Grungberg 1978, Hadidy 2012, Kachouchi 2018, Laajasalo 2005, Large 2008, Leong 1995, Liem 2012, Meehan 2006, Millaud 1989, Nielssen 2007, Nielssen 2010, Nielssen 2011, Nielssen 2011, Nielssen 2012, Nielssen 2012, Nielssen 2022, Nordström 2006, Penney 2023, Provoost 2022, Simpson 2006, Valença 2008, Valevski 1999, Wang 2019, Yee 2011	Meta-analytic findings indicate 38.5% (95% CI 31.1%−46.5%) of homicide offenders with psychosis are early psychosis [35, 46, 54, 57, 64, 74, 78, 89, 96]. Findings from additional studies estimate that 12%−70% of homicide/severe violence offenders with psychosis/severe mental illness are early psychosis [39, 41, 45, 51, 53, 58, 63, 67, 70, 75, 77, 79, 80, 82, 84, 85, 87, 94, 97, 99, 100]. Rates of homicide are 6–15 times higher in early psychosis relative to previously treated psychosis [35, 55]. Early psychosis patients committing homicide/severe violence are younger, more likely to come from non-English speaking countries and to have used a firearm in the offense relative to patients with previously treated psychosis [80]. The proportion of people committing stranger homicide is not significantly different in early psychosis versus previously treated psychosis (64% vs. 52%, respectively) [81]. Longer duration of untreated psychosis at the country level is associated with a higher proportion of early psychosis patients who commit homicide, relative to previously treated psychosis [65]. Among forensic patients who committed homicide and are experiencing a first episode of psychiatric illness, 95% were early psychosis [42]. Case studies suggest that delusional misidentification syndromes may be associated with homicide/violence in early psychosis [43, 48].
Domestic homicide/filicide/parricide	10 (14%)	Ali 2024, Bruwer 2017, Carabellese 2014, LeBihan 2004, Lewis 2003, Markopoulou 2024, Nielssen 2009, Petroni 2022, Valença 2011, Valença 2021	Prevalence of early psychosis among offenders hospitalized for a psychiatric assessment in South Africa following domestic homicide was 60% [49]. Prevalence of early psychosis among men hospitalized due to parricide in a French hospital was 38% [66]. A study from Greece found no significant difference in the proportion of parricide offenders vs. homicide offenders who were early psychosis at the time of offence (29% vs. 39%, respectively) [101]. Prevalence of early psychosis cases among offenders committing child homicide with a psychotic illness was 58% in the Australian state New South Wales [76]. Among women with psychosis committing filicide and undergoing psychiatric assessment, 14% were early psychosis. Among all women undergoing psychiatric assessment for filicide in the state of Michigan, 9% were early psychosis [69]. Case studies suggest that specific types of delusions (persecutory delusions and misidentification [eg, Capgras’ delusion]) were associated with parricide and filicide in early psychosis [50, 86, 93, 95, 102], as well as other factors such as peripartum onset [102], limited social support, family conflict and auditory hallucinations [93].
Violence	3 (5%)	Davies 2006, He 2022, Tengstrom 2001	Among male offenders with schizophrenia convicted of a violent offense, 22% were early psychosis. There were no differences in the prevalence of early psychosis between the early and late start offender groups with schizophrenia (23% vs. 22%) [92]. Among patients with schizophrenia undergoing forensic psychiatric assessment for interpersonal violence, 26% were early psychosis [60]. A case study suggests powerlessness (lack of control) may contribute to violence in early psychosis [52].
Sex offences	2 (3%)	Alish 2007, Smith 1999	There was a higher proportion of early psychosis in the schizophrenia-sex offences group relative to the schizophrenia non-sex-offence group (28% vs. 13%; all men) [40]. Among men with schizophrenia hospitalized for sex offences, 10% were early psychosis. There was a higher proportion of early psychosis patients in the white group vs. Black-Caribbean (29% vs. 5%) [91].

Among psychosis/severe mental illness groups hospitalized or assessed for committing domestic homicide, filicide, or parricide, early psychosis prevalence ranged from 14% to 60% (Table 3) [49, 66, 69, 76, 101]. One study found no difference in early psychosis prevalence between parricide and other homicide groups [101]. A review found that longer duration of untreated psychosis was associated with higher homicide risk in early psychosis [65]. Two sex-offence studies reported 10% and 28% of male schizophrenia inpatients hospitalized for a sex offence were early psychosis [40, 91]. Case studies highlighted features associated with homicide/violence (and resulting forensic mental health service use), including persecutory delusions, delusional misidentification syndromes [43, 48, 50, 86, 93, 95, 102], feelings of powerlessness [52], and intellectual and developmental disabilities [56].

### Forensic mental health services population and other evidence sources

Sixteen studies focused on forensic mental health services populations (22%), and two did not fit population categories (3%; Table 4). Two studies reported early psychosis prevalence of 5% [98] and 8% [83] among all forensic psychiatric inpatients. Among forensic inpatients with psychotic disorders, specifically, early psychosis prevalence ranged from 22% to 40% [61, 72, 103–106]. Three studies found that early psychosis was significantly more common among forensic inpatients compared to non-offender inpatients [104–106]. In offenders undergoing psychiatric assessment, early psychosis prevalence was 24% in a sample with schizophrenia [44], and 60% in a sample with delusional disorder [107]. Other forensic mental health services populations included an outpatient early intervention forensic clinic for severe mental illness, in which 40% were early psychosis [88]. Another study of a forensic outpatient sample with no prior psychosis diagnosis found that 9% screened positive for early psychosis [108]. The two sources in the “other” category included a case vignette providing clinical guidance on psychological intervention for youth with early psychosis in forensic settings [90], and a column highlighting the role of forensic mental health services in diverting people with early psychosis from incarceration [37].

**Table 4:** Summary of evidence regarding early psychosis in forensic populations (*N* = 16) and other sources of evidence (*N* = 2)

Evidence population category	n (% of all included studies, *N*=74)	Studies	Summary of evidence
Forensic inpatients	12 (16%)	Goodhand 2024, Gralton 2000, Hardwick 2003, Hodgins 2004, Jacobshagen 2024, Kirchebner 2023, Machetanz 2023, Markopoulou 2021, Markopoulou 2023, Nijman 2003, Singh 2024, Weithmann 2008	In samples of forensic inpatients, 5% [98] and 8% [83] were early psychosis. Patients whose first ever psychiatric admission was to medium security accounted for 5% of all admissions, and early psychosis cases accounted for most of those first ever admissions (75%−85%) [59]. Among patients with psychosis or schizophrenia admitted to a forensic hospital/found not guilty due to mental illness, early psychosis cases account for 22% to 40% of patients [61, 72, 103–106]. Three studies found a significantly higher proportion of early psychosis cases in an offender inpatient group versus non-offender inpatients with schizophrenia spectrum disorder [104–106]. A Greek study comparing early psychosis forensic inpatients with a previously treated psychosis group found that early psychosis patients were younger, more likely to have recently experienced a stressful life event, and more likely to have assaulted a family member [72, 109]. A history of conduct disorder was not associated with committing an offence during early psychosis versus the course of illness [110]. Another study comparing men with early schizophrenia to those with prior admissions, found that there were no differences between groups in age and proportion of first-generation migrants [61]. A case series suggests that schizophrenia may go unrecognized in forensic samples of people with mild and borderline learning disability, and may explain violent offending behaviour [56].
Forensic outpatients	2 (3%)	Koks 2024, Purcell 2012	In a sample of youth referred to a pilot forensic clinic embedded within early intervention services for mental illness, 40% were early psychosis. [88] In sample of forensic outpatients with no prior diagnosis of psychotic disorder and screened for psychosis symptoms, 9% were found to be early psychosis [108].
Forensic psychiatric assessments	2 (3%)	Bègue 2020, Boylu 2025	24% of offenders with schizophrenia undergoing psychiatric assessment were early psychosis. There were no differences in the proportion of early psychosis in the comorbid substance use disorder group vs. no substance use disorder (27% vs. 21%) [44]. In a sample of patients with delusional disorder undergoing forensic psychiatric assessment, 60% were early psychosis. There was no significant difference in the proportion of people with early psychosis in the group with crime history vs. no crime history (57% vs. 60%, respectively) [107].
Other sources	2 (3%)	Smedley 2010, Wasser 2017	A case vignette illustrates the use of psychological intervention with a young person experiencing a first episode of psychosis in a secure hospital setting, as an example for clinicians [90]. Forensic evaluations are an intercept point along the sequential intercept model in which to divert people experiencing early psychosis from incarceration [37].

## Discussion

We identified a substantial body of literature (74 sources) examining the co-occurrence of forensic mental health services and early psychosis. Most evidence came from cross-sectional studies reporting the prevalence of early psychosis among people with psychosis contacting forensic mental health services because of committing homicide/severe violence, including three systematic reviews on this topic. Findings suggest a high prevalence of early psychosis among homicide/severe violence offenders with psychosis and that this phase of illness is a period of high-risk relative to chronic psychosis. Other populations studied in the literature included people with psychosis committing violence or sex offences, forensic inpatients, those undergoing forensic psychiatric assessments, and outpatient forensic mental health services populations. Early psychosis prevalence was generally lower in broader forensic mental health services samples than in those specific to homicide/severe violence. Few studies examined forensic mental health service use within early psychosis samples. Within the five studies focusing on an early psychosis population, three reported on either the incidence of forensic mental health service use or associated risk factors, and one assessed health service use outcomes following forensic hospitalization.

Interpretation of findings in this review is complicated by how “early psychosis” was defined across studies. We found the concept of “early psychosis” was either not clearly delineated in most studies, or the definitions used were associated with limitations. For studies where early psychosis was not clearly defined, this is because these studies were not concerned with examining “early psychosis” cases *per se*, and instead reported characteristics correlated with early psychosis, such as lack of prior treatment or contact with psychiatric services. Importantly, prior contact definitions for early psychosis may miss cases [34], resulting in misclassification of some early psychosis patients as chronic. Therefore, prevalence estimates of early psychosis within different forensic mental health services populations reported in this review may be underestimated. Two studies focusing on incidence of forensic hospitalization in early psychosis cohorts used first hospitalization criteria to identify early psychosis cases. In these studies, cohorts are missing early psychosis cases from the denominator who were never hospitalized, approximately half of early psychosis cases [5], suggesting the incidence estimates for forensic hospitalization may be over-estimated.

Most evidence on forensic mental health service use and early psychosis came from offence-specific studies, mainly focusing on homicide. These studies used contact with forensic mental health services to define the study population, including groups hospitalized, found not guilty due to mental illness, or undergoing forensic psychiatric assessment for committing homicide. While these studies were included in our review, they were not designed to characterize forensic mental health service use but instead aimed to examine homicide in people with psychosis. As a result, the evidence base on forensic mental health service use and early psychosis disproportionately reflects this small, but high-risk subgroup, while overlooking broader forensic mental health services populations. Taken together with the finding that most studies were also not focused on early psychosis *per se*, few studies in this review directly aimed to examine both an early psychosis group and forensic mental health services. As a result, our understanding of early psychosis populations within forensic mental health services, such as prevalence and characteristics of this group, remains limited.

While broad, recent evidence suggests that people with psychosis have elevated risks of violence [27, 28], our review further highlights that early psychosis is a high-risk period for forensic mental health service involvement as a result of committing homicide/severe violence, relative to chronic psychosis [35]. We additionally found that studies of broader forensic inpatient populations generally reported a lower prevalence of early psychosis [83, 98] than studies examining forensic mental health service users who committed homicide [35]. This may suggest that while early psychosis is a high-risk period for hospitalization for committing homicide/severe violence, forensic psychiatric inpatients as a whole include more people with chronic psychosis and other mental disorders. This may reflect prolonged hospital stays typical in forensic inpatient settings beyond the early phase of illness. However, interpretation of prevalence estimates reported in this review are complicated by international variation in mental health legislation and the scope of forensic mental health services across countries [111, 112]. Such variation contributes to heterogeneity in cases included in the forensic mental health services population denominators across jurisdictions, limiting comparability.

### Future directions

There is substantial cross-sectional evidence indicating that many homicide offenders in forensic mental health services are in the early phase of psychosis. Further research aimed at directly examining both early psychosis and forensic mental health services more broadly would provide better evidence on the prevalence in forensic mental health settings, as well as incidence and risk factors for forensic mental health services involvement in early psychosis. This is essential information for planning and implementing interventions for those in the early phase of illness in forensic mental health services or at risk of entering forensic mental health services. Operationalization of early psychosis using duration of illness criteria would provide more consistency and help clarify the true prevalence of early psychosis within forensic mental health services. There is also a need for more research focused on early psychosis cohorts to better understand the frequency and nature of forensic mental health service use, as well as associated factors to identify high-risk subgroups in general psychiatric services. Future studies should also investigate broader outcomes following forensic hospitalization in early psychosis beyond rehospitalization, such as clinical and social functioning, engagement in psychiatric services, and recidivism to better understand the impact of forensic mental health service use during this period of illness. It would also be informative to characterize outpatient forensic mental health service populations, such as those undergoing outpatient assessments, as most studies focus on inpatients. Most studies were conducted between 1990 and 2009, with many covering earlier time periods. Considering the growing demand for forensic mental health service provision [25] and the international expansion of early psychosis intervention services through the 2000s [3, 113], more recent evidence is needed. We also observed regional gaps in evidence, with most studies conducted in Europe and few studies from Africa and South America. More evidence is needed from these regions.

### Limitations of this review

As this was a scoping review, we did not assess the quality or risk of bias of included studies. Studies differed in design, early psychosis case ascertainment, and timeframe. More thorough investigation and evidence synthesis including risk of bias assessment alongside relevant stratifications would be useful for better assessing this literature. Stratifying evidence by timeframe may be particularly important, given changes in both mental health and forensic service provision over time and changes in legislation, which would impact pathways into forensic mental health services and case definitions. Our inclusion criteria relied on health service-based definitions of early psychosis, such as first contact with mental health services, and are likely overly conservative [34]. As a result, some evidence reported in this review likely excluded some early psychosis cases.

## Conclusions

Most available evidence on the overlap of forensic mental health services and early psychosis comes from cross-sectional studies of people with severe mental illness who committed homicide, though many of these studies did not directly aim to study either forensic mental health service use or early psychosis *per se*. While early psychosis appears to be a period of elevated risk for homicide, there is limited evidence examining forensic mental health service use in early psychosis populations. Evidence available suggests that up to 1 in 20 people with early psychosis experience forensic hospitalization. Evidence also suggests that men (Black-Caribbean men, in particular) and those with prior police contact have higher risk of forensic hospitalization during this period, and that experiencing forensic hospitalization in early psychosis is associated with intensive long-term inpatient service use. Further research is needed to better characterize the frequency and nature of contact with forensic mental health services in early psychosis, as well as associated factors and outcomes, from a broader health system perspective.

## Supplementary Information

Below is the link to the electronic supplementary material.

Supplementary Material 1

Supplementary Material 2

## Data Availability

Data available upon request.
