# Supplementary material for: Forensic mental health service use in early psychosis: A scoping review
Source: Soc Psychiatry Psychiatr Epidemiol. 2026 Jan 19;61(10):1981–96. doi: 10.1007/s00127-025-03038-4 (PMC13585902; doi:10.1007/s00127-025-03038-4)
Supplement: Supplementary file 2 [file 127_2025_3038_MOESM2_ESM.pdf]

Table S1. Preferred Reporting Items for Systematic reviews and Meta-Analyses extension for Scoping Reviews (PRISMA-ScR) Checklist.

SECTION	ITEM	PRISMA-ScR CHECKLIST ITEM	REPORTED ON PAGE #
**TITLE**			
Title	1	Identify the report as a scoping review.	1 (title page document)
**ABSTRACT**			
Structured summary	2	Provide a structured summary that includes (as applicable): background, objectives, eligibility criteria, sources of evidence, charting methods, results, and conclusions that relate to the review questions and objectives.	1
**INTRODUCTION**			
Rationale	3	Describe the rationale for the review in the context of what is already known. Explain why the review questions/objectives lend themselves to a scoping review approach.	3-4
Objectives	4	Provide an explicit statement of the questions and objectives being addressed with reference to their key elements (e.g., population or participants, concepts, and context) or other relevant key elements used to conceptualize the review questions and/or objectives.	4
**METHODS**			
Protocol and registration	5	Indicate whether a review protocol exists; state if and where it can be accessed (e.g., a Web address); and if available, provide registration information, including the registration number.	4
Eligibility criteria	6	Specify characteristics of the sources of evidence used as eligibility criteria (e.g., years considered, language, and publication status), and provide a rationale.	4-5
Information sources*	7	Describe all information sources in the search (e.g., databases with dates of coverage and contact with authors to identify additional sources), as well as the date the most recent search was executed.	6
Search	8	Present the full electronic search strategy for at least 1 database, including any limits used, such that it could be repeated.	Supplemental table S2
Selection of sources of evidence†	9	State the process for selecting sources of evidence (i.e., screening and eligibility) included in the scoping review.	6
Data charting process‡	10	Describe the methods of charting data from the included sources of evidence (e.g., calibrated forms or forms that have been tested by the team before their use, and whether data charting was done independently or in duplicate) and any processes for obtaining and confirming data from investigators.	6-7
Data items	11	List and define all variables for which data were sought and any assumptions and simplifications made.	6-7

SECTION	ITEM	PRISMA-ScR CHECKLIST ITEM	REPORTED ON PAGE #
Critical appraisal of individual sources of evidence§	12	If done, provide a rationale for conducting a critical appraisal of included sources of evidence; describe the methods used and how this information was used in any data synthesis (if appropriate).	N/A
Synthesis of results	13	Describe the methods of handling and summarizing the data that were charted.	7
**RESULTS**			
Selection of sources of evidence	14	Give numbers of sources of evidence screened, assessed for eligibility, and included in the review, with reasons for exclusions at each stage, ideally using a flow diagram.	7, Figure 1
Characteristics of sources of evidence	15	For each source of evidence, present characteristics for which data were charted and provide the citations.	7-8, Table 1, Table S3
Critical appraisal within sources of evidence	16	If done, present data on critical appraisal of included sources of evidence (see item 12).	N/A
Results of individual sources of evidence	17	For each included source of evidence, present the relevant data that were charted that relate to the review questions and objectives.	8-11, Tables 1-3, Table S4
Synthesis of results	18	Summarize and/or present the charting results as they relate to the review questions and objectives.	8-11, Tables 1-3
**DISCUSSION**			
Summary of evidence	19	Summarize the main results (including an overview of concepts, themes, and types of evidence available), link to the review questions and objectives, and consider the relevance to key groups.	11
Limitations	20	Discuss the limitations of the scoping review process.	15
Conclusions	21	Provide a general interpretation of the results with respect to the review questions and objectives, as well as potential implications and/or next steps.	15-16
**FUNDING**			
Funding	22	Describe sources of funding for the included sources of evidence, as well as sources of funding for the scoping review. Describe the role of the funders of the scoping review.	16

PRISMA-ScR = Preferred Reporting Items for Systematic reviews and Meta-Analyses extension for Scoping Reviews. *From:* Tricco AC, Lillie E, Zarin W, O'Brien KK, Colquhoun H, Levac D, et al. PRISMA Extension for Scoping Reviews (PRISMAScR): Checklist and Explanation. Ann Intern Med. 2018;169:467–473. doi: 10.7326/M18-0850.

* Where *sources of evidence* (see second footnote) are compiled from, such as bibliographic databases, social media platforms, and Web sites.

† A more inclusive/heterogeneous term used to account for the different types of evidence or data sources (e.g., quantitative and/or qualitative research, expert opinion, and policy documents) that may be eligible in a scoping review as opposed to only studies. This is not to be confused with *information sources* (see first footnote).

‡ The frameworks by Arksey and O'Malley (6) and Levac and colleagues (7) and the JBI guidance (4, 5) refer to the process of data extraction in a scoping review as data charting.

§ The process of systematically examining research evidence to assess its validity, results, and relevance before using it to inform a decision. This term is used for items 12 and 16 instead of "risk of bias" (which is more applicable to systematic reviews of interventions) to include and acknowledge the various sources of evidence that may be used in a scoping review (e.g., quantitative and/or qualitative research, expert opinion, and policy document).

Table S2. Search strategy used in MEDLINE.

Concept	Step	Search
Psychosis	1	affective disorders, psychotic/ or psychotic disorders/ or exp schizophrenia/
	2	(psychosis or psychoses or psychotic or schizophreni* or sever* mental* ill* or severe mental disorder*).ti,ab.
First-episode or early psychosis	3	(((early or first or recent) adj3 (psychosis or psychotic or psychoses or schizophreni* or episode* or intervention)) or (first adj3 (hospital* or admit* or admission*))).ti,ab.
Forensic mental health services	4	exp Forensic Psychiatry/
	5	exp Mental Competency/
	6	(((forensic or secur*) adj3 (patient* or inpatient* or detainee* or admission* or admit* or hospital* or ward* or unit* or facilit* or institute or order*)) or (forensic adj3 (mental health or psychiatr*)) or ((low or medium or high or maximum) adj3 secur*) or (insanity acquittee* or insanity defen* or offender patient* or mentally ill offender* or mental illness defen* or "not criminally responsible" or "unfit to stand trial" or "not guilty by reason of insanity")).mp
	7	1 or 2
	8	3 and 7
	9	4 or 5 or 6
	10	8 and 9

Table S3. Characteristics of each included source of evidence (N = 74).

Author, year	Publication type	Country	Study aim(s)	Setting	Study design	Time-frame	Data sources	Population	N	Early psychosis definition	Forensic concept definition
Ali 2024 [1]	Abstract	United States	Inform the clinical picture and the legal implications associated with postpartum psychosis, and to emphasize the importance of considering other psychiatric illnesses with peripartum onset that affect maternal and pediatric population wellbeing	One emergency department	Case study	Not reported	Clinical observation	Case of a female in her 20s, mother of two toddlers, with a history of posttraumatic stress disorder and postpartum depression	1	No prior psychiatric treatment for psychosis	Admitted to forensic hospital or unit
Alish 2007 [2]	Journal article	Israel	Investigate clinical, socio-demographic and sexual factors associated with deviant sexual behavior in sex offenders with schizophrenia	One forensic hospital	Cross-sectional	2000-2004	Hospital chart review	Adult males with a diagnosis of schizophrenia and hospitalized for sex offence or any other criminal activity; comparison group: hospitalized population over the study period for sex offences but no schizophrenia. Brain damage or mental retardation excluded.	173	No prior hospitalization for psychosis	Admitted to forensic hospital or unit
Amani 2022 [3]	Journal article	Algeria	Describe characteristics of patients with schizophrenia who commit homicides, of their victims and of the methods and circumstances of their homicides compared to those committed by people without serious mental disorders	One forensic hospital (covering Western region of Algeria)	Cross-sectional	2015-2018	Medical records, information from patients and families	All patients with schizophrenia judicially interned for homicide	60	No prior psychiatric treatment for psychosis	Admitted to forensic hospital or unit
Azoulay 2020 [4]	Journal article	France	Determine whether homicide primarily	One forensic hospital	Cross-sectional	2009-2018	Medical and criminal records	Patients with no history of psychiatric care	19	No prior psychiatric	Admitted to forensic

Author, year	Publication type	Country	Study aim(s)	Setting	Study design	Time-frame	Data sources	Population	N	Early psychosis definition	Forensic concept definition
			occurs during the first episode of psychosis					admitted following homicide or an attempted homicide		treatment for psychosis	hospital or unit
Balcioglu 2024 [5]	Journal article	Turkiye, Italy	Conduct a comparative analysis of homicide perpetrators with psychotic illnesses in Italy and Turkiye, identifying the extent to which country-specific factors influence offender profiles and crime-scene characteristics	Two forensic psychiatric centres	Cross-sectional	2018-2023	Chart review, medico-legal records	Subjects who had been assessed for criminal responsibility for the index homicide offense at both units (Italy and Turkey) from January 1, 2018, to January 1, 2023 with a confirmed diagnosis of a DSM-5 SSD established during forensic psychiatric evaluations	67	No prior contact with mental health services for psychosis	Forensic psychiatric assessment
Barbieri 2022 [6]	Journal article	Italy	Present the case of homicide as a result of the Doppelganger phenomenon	Not reported	Case study	Not reported	Not reported	Case study involving Doppelganger phenomenon and homicide	1	No prior psychiatric treatment for psychosis	Forensic psychiatric assessment
Bègue 2020 [7]	Journal article	France	Assess the frequency of substance use disorders in people with schizophrenia who had committed criminal offenses and compare the socio-demographic, historical, clinical, and criminal characteristics of these subjects with and without an associated substance use disorder	Not reported	Cross-sectional	2001-2018	Files from forensic psychiatric assessments	All forensic psychiatric reports involving patients with schizophrenia	104	No prior contact with mental health services for psychosis	Forensic psychiatric assessment
Belli 2010 [8]	Journal article	Turkey	Describe social and clinical characteristics and offense details in a sample of people with schizophrenia convicted of homicide	One forensic hospital covering eastern region of Turkey	Cross-sectional	2004-2007	Data collected from interviews with patients, family members or review of hospital records	People with schizophrenia convicted of homicide and hospitalized in the forensic psychiatry unit; severe organic disease or cognitive impairment excluded.	49	No prior contact with mental health services for psychosis	Admitted to forensic hospital or unit

Author, year	Publication type	Country	Study aim(s)	Setting	Study design	Time-frame	Data sources	Population	N	Early psychosis definition	Forensic concept definition
Böker & Häfner 1982 [9]	Other: Book	Germany	Examine the offender-incidence rates for dangerous crimes committed by the mentally ill, compared with the general population and identify risk factors; investigate the motivation for the crime and its relationship to the illness; describe the circumstances of the crime in the mentally ill in relation to the groups of illness, offender sex, and other variables	Population-based (national coverage)	Cross-sectional	1955-1964	Federal statistics of convicted offenders, court files, medical files	Mentally ill violent offenders (homicide or attempted homicide) whose crimes were committed between 1 January 1955 to 31 December 1964.	533	No prior hospitalization for psychosis	Found not guilty due to mental illness; Found unfit to stand trial; Forensic psychiatric assessment
Bourget 2004 [10]	Journal article	Canada	Describe demographic, psychiatric and social characteristics of a sample of murderers found to suffer from first-episode psychosis	Not reported	Case series	Not reported	Records from forensic psychiatric examination	Cases referred for a forensic psychiatric exam charged with homicide or attempted homicide and diagnosed with a first psychotic episode after the homicidal event.	22	No prior psychiatric treatment for psychosis	Forensic psychiatric assessment
Bourget 2004 [11]	Journal article	Canada	Review the neurophysiological correlates and clinical factors observed in cases of Capgras syndrome, and present and discuss commonly reported demographic and clinical features that may contribute to an increased risk for violence	Not reported	Case series; Narrative review	Not reported	Clinical interviews, review of medical charts, crown brief synopses, hearings, court transcripts, and newspaper reports	Not reported	4	No prior psychiatric treatment for psychosis	Forensic psychiatric assessment
Boylu 2025 [12]	Journal article	Turkey	Examine the sociodemographic and clinical characteristics of patients with delusional disorder who have committed crimes, with a	One forensic department	Case-control	2018-2022	Medico-legal records	Cases diagnosed with delusional disorder who underwent forensic psychiatric evaluation by the Expertise Department of	150	No prior psychiatric treatment for psychosis	Forensic psychiatric assessment

Author, year	Publication type	Country	Study aim(s)	Setting	Study design	Time-frame	Data sources	Population	N	Early psychosis definition	Forensic concept definition
			particular focus on the factors that contribute to criminal behaviour and criminal characteristics					Psychiatric Observation and 4th Specialised Board of the Council of Forensic Medicine between 1 January 2018 and 31 December 2022; a control group was formed of patients with delusional disorder but no criminal history, who had been sent for guardianship evaluation by the civil courts			
Bruwer 2017 [13]	Dissertation	South Africa	Investigate domestic homicides by offenders with severe mental illness referred to the Forensic Mental Health Service at Valkenberg Hospital for forensic psychiatric observation.	One forensic hospital, covering all forensic psychiatric observations in the Western Cape	Cross-sectional	2010-2014	Medical files	All persons charged with domestic homicide that were referred for assessment and found to have a lifetime diagnosis of severe mental illness	21	No prior contact with mental health services for psychosis	Admitted to forensic hospital or unit; Forensic psychiatric assessment
Carabellese 2014 [14]	Journal article	Italy	To better understand the relationship between Capgras syndrome and violence	Not reported	Case study	Not reported	Not reported	Cases of matricide by sons with psychosis in which involved delusional misidentification	2	No prior psychiatric treatment for psychosis	Forensic patient
Carabellese 2021 [15]	Journal article	Italy	Describe the psychiatric and criminalistic characteristics of a forensic psychiatric population of defendants who were charged with homicide, who at the conclusion of a forensic psychiatric examination had been recognized as suffering from schizophrenia spectrum disorder or not at the time of the murder.	Forensic assessments done by one forensic hospital	Cross-sectional	10-year period, years not reported	Medical files from forensic psychiatric assessments and procedural documentation	Cases of homicide or attempted homicide in which defendant underwent a forensic psychiatric evaluation over the previous 10 years	187	No prior hospitalization for psychosis	Forensic psychiatric assessment

Author, year	Publication type	Country	Study aim(s)	Setting	Study design	Time-frame	Data sources	Population	N	Early psychosis definition	Forensic concept definition
Chen 2018 [16]	Journal article	China	Describe differences between alleged homicide offenders with and without psychosis	Forensic Medical Service covering province of Sichuan	Cross-sectional	1998-2006	Medical and criminal records	Homicide cases referred for psychiatric assessment	210	No prior psychiatric treatment for psychosis	Found not guilty due to mental illness; Forensic psychiatric assessment
Davies 2006 [17]	Journal article	Australia	Present two cases of first episode schizophrenia in which the fact or risk of violence was a major issue in presentation and management	Not reported	Case study	Not reported	Not reported	Two cases (one who committed an act of violence and the other with violent ideation), undiagnosed at time of index event and presented for medicolegal reports within a 2-year period	2	No prior psychiatric treatment for psychosis; Other: Undiagnosed at time of violent index event	Forensic psychiatric assessment
Dolan 1996 [18]	Journal article	United Kingdom	Describe and compare demographic, criminological, and psychiatric details of those committing psychotic and non-psychotic homicides	One forensic hospital	Cross-sectional	August 1 1994	Medical case records, court reports, witness statements	All male inpatients on August 1, 1994, whose index offence was homicide	120	No prior contact with mental health services for psychosis	Admitted to forensic hospital or unit
Erb 2001 [19]	Journal article	Germany	Compare 1) the proportions of homicides committed by persons with schizophrenia, 2) the proportions of persons with schizophrenia who committed homicide, and 3) the characteristics of the homicide offenders with schizophrenia in a historical vs recent time period (1955-1964 and 1992-1996)	Period 1: population-based in Federal Republic of Germany; Period 2: One forensic hospital covering the state of Hessen (population-based)	Cross-sectional	1955-1964 and 1992-1996	Medico-legal files	All persons with schizophrenia who had committed or attempted to commit homicide in each region and time period	313	No prior hospitalization for psychosis	Admitted to forensic hospital or unit
Golenkov 2011 [20]	Journal article	Russia	Describe demographic, clinical and criminological characteristics of	Chuvashia region (one hospital/outpatients in prison)	Cross-sectional	1981-2010	Psychiatric reports, hospital records,	All homicide offenders in Chuvashia suspected of having a psychiatric disorder referred for	133	No prior psychiatric treatment for psychosis	Forensic psychiatric assessment

Author, year	Publication type	Country	Study aim(s)	Setting	Study design	Time-frame	Data sources	Population	N	Early psychosis definition	Forensic concept definition
			homicide offenders with schizophrenia in Chuvash Republic				outpatient notes, legal documents	psychiatric examination and found to have schizophrenia			
Goodhand 2024 [21]	Journal article	Australia	Establish the patterns of contact with health services among forensic patients and to identify the sociodemographic and clinical characteristics associated with lack of contact or effective intervention	New South Wales, Australia	Cohort	1990-2016	Medico-legal records linked with health administrative data	Forensic patients found not guilty of a serious offence on the grounds of mental illness in New South Wales, Australia between Jan 1 1990 and Jul 29 2016 and where the index offence was at least 6 months after the start of each relevant health dataset	307	No prior contact with mental health services for psychosis	Found not guilty due to mental illness
Gralton 2000 [22]	Journal article	England	Discuss case-histories of previously unrecognized schizophrenia in six male patients with mild and borderline-learning disability admitted to a forensic unit	Forensic hospital unit with catchment covering two counties in south-west England	Case series	Not reported	Not reported	Six male patients admitted to an open forensic psychiatric unit for people with mild and borderline learning disability, found to have previously unrecognized schizophrenia	6	No prior psychiatric treatment for psychosis; Other: Previously unrecognized psychosis	Admitted to forensic hospital or unit
Grungberg 1978 [23]	Journal article	United States	Examine whether the shift in community-based psychiatric services is associated with an increase in homicide in people with a history of mental illness or found not guilty by reason of insanity	Population-based (Albany County, New York)	Case study; Cross-sectional; Other: Pre-post study	1963-1975	Records from District Attorney's office	All individuals arrested for homicide and convicted of first- or second-degree murder or non-negligent manslaughter, or acquitted by reason of insanity	48	No prior hospitalization for psychosis	Found not guilty due to mental illness
Hadidy 2012 [24]	Journal article	Saudi Arabia	Examine the socio-demographic and clinical differences among patients with schizophrenia who were convicted of homicide and patients with schizophrenia who did not attempt homicide	One forensic hospital, outpatient clinic	Cross-sectional; Case-control	2008-2009	Survey/interview of patients	Patients with schizophrenia (15-80 years of age) who committed homicide and referred for forensic examination and general psychiatry patients who did not commit homicide (matched on age and sex, from the same	246	No prior hospitalization for psychosis; Other: "First episode psychotic illness" (experiencing psychosis for the first time)	Forensic psychiatric assessment

Author, year	Publication type	Country	Study aim(s)	Setting	Study design	Time-frame	Data sources	Population	N	Early psychosis definition	Forensic concept definition
								hospital). Exclusions: refusal to give consent, IQ below 70, organic brain disorder, clinical language or hearing disability.			
Hardwick 2003 [25]	Journal article	United Kingdom	Describe the characteristics of a group of patients admitted to a Medium Security Unit over the course of a ten-year period who had never previously had any form of psychiatric inpatient care	One medium secure unit	Cross-sectional	1988-1997	Register database	All patients admitted from 1988 to 1997	626	No prior psychiatric treatment for psychosis	Admitted to forensic hospital or unit
He 2022 [26]	Journal article	China	Compare demographic, clinical, and criminological characteristics of the patients with schizophrenia who committed different types of violence to relatives, acquaintances, or strangers	One forensic psychiatric assessment centre	Cross-sectional	2015-2019	Medical and law enforcement records, patient assessment records	Forensic inpatients with schizophrenia (and no comorbidities), history of interpersonal violence, and complete case data	332	No prior psychiatric treatment for psychosis	Admitted to forensic hospital or unit; Forensic psychiatric assessment
Hodgins 2004 [27]	Journal article	Canada, Finland, Germany, Sweden	Identify opportunities for interventions to prevent offending among men with schizophrenia by tracking their histories of offending and admissions to hospital	Four forensic psychiatric hospitals (one in each country), and general psychiatric hospitals in the same geographical region	Cross-sectional	Not reported	Patient and family interviews, school, medical, social service, and criminal files	Consecutively discharged male patients with a diagnosis of schizophrenia spectrum disorder who had received inpatient care from the forensic psychiatric hospital in one of the four sites. For each forensic patient recruited, a patient from a general psychiatric hospital in the same geographical region of the same gender and age (+/- 5 years) and	232	No prior hospitalization for psychosis	Admitted to forensic hospital or unit

Author, year	Publication type	Country	Study aim(s)	Setting	Study design	Time-frame	Data sources	Population	N	Early psychosis definition	Forensic concept definition
								with the same diagnosis was included.			
Humphreys 1994 [28]	Journal article	United Kingdom	Describe offending behaviour in patients in their first episode of schizophrenia who committed an offence within 5 years prior to first admission	One hospital and several medical centres within 35 miles of the London borough of Harrow	Cross-sectional	Not reported	Interview with patients, families, and nursing staff	Patients with a first episode of schizophrenia (15-70 years old) with no previous hospitalization and offending history within 5 years prior to first admission	31	No prior psychiatric treatment for psychosis	Admitted to forensic hospital or unit
Jacobshagen 2024 [29]	Journal article	Switzerland	Investigate the difference between offender female patients and non-offender female patients with schizophrenia spectrum disorder	One forensic hospital and one general psychiatry hospital	Cross-sectional	1982-2016	Medical records, police and court reports and testimonies	Female patients admitted to the forensic hospital between 1982 and 2016 with an ICD-9 or ICD-10 diagnosis of SSD and female non-offenders with SSD admitted to the general psychiatry hospital	60	No prior psychiatric treatment for psychosis; No prior contact with mental health services for psychosis; No prior hospitalization for psychosis	Admitted to forensic hospital or unit
Kachouchi 2018 [30]	Journal article	Morocco	Describe characteristics of homicide in patients suffering from schizophrenia and to determine the correlated sociodemographic and clinical variables	One psychiatric hospital	Cross-sectional	2005-2015	Review of files, patient interview, patient survey	Patients with diagnosis of schizophrenia hospitalized at the Ibn Nafis university psychiatric hospital	120	No prior psychiatric treatment for psychosis	Admitted to forensic hospital or unit
Kirchebner 2023 [31]	Journal article	Switzerland	Identify key differences in offender patients and non-offender patients regarding aggressive behavior through application of supervised machine learning, and quantify the performance of the model	One forensic hospital and one general psychiatric hospital	Cross-sectional	1982-2016	Medical and legal case files	Patients admitted to the forensic hospital between 1982 and 2016 with a diagnosis of SSD and patients admitted to the general psychiatry hospital with a diagnosis of SSD matched on gender	740	No prior contact with mental health services for psychosis; No prior hospitalization for psychosis	Admitted to forensic hospital or unit
Koks 2024 [32]	Journal article	Netherlands	Establish the prevalence of ultra-high risk (UHR) for psychosis among a forensic population and	Forensic psychiatric outpatient setting	Cross-sectional	Not reported	Patient interview/ assessment	Patients of a forensic psychiatric outpatient setting with no prior diagnosis or treatment for psychotic symptoms	76	No prior psychiatric treatment for psychosis	Forensic patient; Other: Forensic psychiatric

Author, year	Publication type	Country	Study aim(s)	Setting	Study design	Time-frame	Data sources	Population	N	Early psychosis definition	Forensic concept definition
			examine its relationship with aggression								outpatient setting
Laajasalo 2005 [33]	Journal article	Finland	Examine early- and late-start homicide offenders with schizophrenia and identify differences in background characteristics and crime scene behaviours	Population-based, national	Cross-sectional	1983-2002	Medico-legal files (forensic examination statements)	People with schizophrenia accused of homicide (murder or manslaughter)	109	No prior contact with mental health services for psychosis	Forensic psychiatric assessment
Large 2008 [34]	Journal article	8 countries in North America, Europe, Middle East	Examine whether there is an association between the average duration of untreated psychosis and the proportion of homicides committed during the first episode of psychosis at the country level	Mixed population-based studies (8 studies) and less complete samples (5 studies did not cover a defined region)	Systematic review	1966-2007	Existing literature	English studies published between 1966 and 2006 reporting groups of people with psychotic illness who were charged with homicide or attempted homicide and included sufficient information to determine whether the subjects had received treatment prior to the homicide. Studies excluded: case reports, small personal series and studies of homicide due to misidentification syndrome or specific types of homicide, no data on DUP of patients from the country available.	13 studies (n=1,361)	No prior psychiatric treatment for psychosis	Found not guilty due to mental illness; Admitted to forensic hospital or unit; Forensic psychiatric assessment
LeBihan 2004 [35]	Journal article	France	Describe men admitted to maximum security hospital for parricide	One forensic hospital	Cross-sectional	1963-2003	Medical files, criminal psychiatric assessments, medicolegal autopsy reports, police reports, dismissal orders	Men who committed parricide and hospitalized to a maximum security	42	No prior hospitalization for psychosis	Admitted to forensic hospital or unit
Leong 1995 [36]	Journal article	United States	Describe a sample of defendants charged with	Psychiatric evaluations	Cross-sectional	1987-1992	Medico-legal records	Psychiatric evaluations performed at the request of the Los Angeles	25	No prior hospitalization for psychosis	Forensic psychiatric assessment

Author, year	Publication type	Country	Study aim(s)	Setting	Study design	Time-frame	Data sources	Population	N	Early psychosis definition	Forensic concept definition
			murder and suffering from psychosis	covering Los Angeles County				County Superior Court, found to be suffering from psychosis			
Levine 2008 [37]	Journal article	Israel	Examine the relationship between legal status at the time of the first psychiatric hospitalization and hospitalization outcomes	National, population-based	Cohort	1978-1992	National psychiatric hospitalization registry	First psychiatric hospitalization for schizophrenia spectrum disorders (1978-1992). Cases with missing data were excluded.	10,591	No prior hospitalization for psychosis	Admitted to forensic hospital or unit
Lewis 2003 [38]	Journal article	United States	Identify differences between psychotic and nonpsychotic filicidal women in relation to their demographic characteristics, history, and offense patterns	One forensic centre, covering all assessments in the state (population-based)	Cross-sectional	1974-1996	Chart review	All cases of women referred to the Center for Forensic Psychiatry for forensic evaluations of competency and/or criminal responsibility for the murder of their biological children	55	No prior hospitalization for psychosis	Forensic psychiatric assessment
Liem 2012 [39]	Journal article	Netherlands	Describe the nature and incidence of homicides committed by people with a psychotic disorder in the Netherlands	Population-based (national coverage)	Cross-sectional	2000-2006	Forensic mental health reports	All Dutch nationals suspected of committing homicide and found to be suffering from a psychotic disorder at the time of the offence	61	No prior psychiatric treatment for psychosis	Forensic psychiatric assessment
Machetanz 2023 [40]	Journal article	Switzerland	Evaluate clinical factors distinguishing offenders and non-offenders with schizophrenia spectrum disorder using supervised machine learning, and to quantify the performance of the calculated machine learning model	One forensic hospital and one general psychiatric hospital	Cross-sectional	1982-2016	Medical and legal files	Forensic psychiatric inpatients with a SSD diagnosis (ICD-10 F2x or ICD-9 295.x) admitted between 1982 and 2016 and a group matched on gender admitted to a general psychiatric hospital with a diagnosis of SSD	740	No prior psychiatric treatment for psychosis; No prior contact with mental health services for psychosis	Admitted to forensic hospital or unit
Macmillan 1987 [41]	Journal article	United Kingdom	Estimate the frequency of police contact in early psychotic illness and the sociodemographic and clinical variables associated; examine the nature and consequences of police	Multi-centre within 35 miles of the Clinical Research Centre, Harrow	Cross-sectional; Cohort	1979-1981	Interviews with patients, relatives, and nursing staff, case-note examination	Patients (15-70 years old) hospitalized for a first psychotic illness, excluding organic psychosis (1979-1981)	253	No prior psychiatric treatment for psychosis	Admitted under forensic sections of the Mental Health Act

Author, year	Publication type	Country	Study aim(s)	Setting	Study design	Time-frame	Data sources	Population	N	Early psychosis definition	Forensic concept definition
			contact, the circumstances in which psychiatric aid was and was not sought, and any relationships between policy contact and outcome of illness								
Markopoulou 2021 [42]	Journal article	Greece	Compare demographic, clinical, and legal characteristics forensic first episode psychosis patients versus previously treated psychosis patients found not guilty by reason of insanity	One forensic hospital covering Northern Greece	Cross-sectional	2015-2020	Medical and court records, interviews with patients, relatives, and ward staff	All patients hospitalized in the Department of Forensic Psychiatry of the Psychiatric Hospital of Thessaloniki with psychotic illness	78	No prior psychiatric treatment for psychosis; Other: Included patients in the first 3 weeks of initiating antipsychotic treatment	Found not guilty due to mental illness
Markopoulou 2023 [43]	Journal article	Greece	Compare demographic, clinical, legal, and psychometric differences in offender patients found not guilty by reason of insanity with conduct disorder versus those without conduct disorder	One forensic hospital covering Northern Greece.	Cross-sectional	2015-2020	Medical records and legal documents, clinical interview with patients, staff interview	Criminal offenders found not guilty by reason of insanity in Northern Greece	78	Other: First psychotic episode	Found not guilty due to mental illness; Admitted to forensic hospital or unit
Markopoulou 2024 [44]	Journal article	Greece	Characterize parricide offenders found not guilty by reason of insanity	One forensic hospital covering Northern Greece	Cross-sectional	2015-2020	Medical and legal records, clinical interview, interview with staff and relatives	Forensic patients hospitalized in Northern Greece between January 2015 and January 2020 found not guilty by reason of insanity for committing homicide, and who consented to participate	52	Other: First psychotic episode	Found not guilty due to mental illness; Admitted to forensic hospital or unit
McGovern 1991 [45]	Journal article	United Kingdom	Compare how Black-Caribbean and white groups enter the psychiatric system, treatment received,	One hospital (Birmingham)	Cohort	1980-1988	Case notes	First psychiatric admission for schizophrenia (1980-1983)	61	No prior hospitalization for psychosis	Admitted under forensic sections of the Mental Health Act

Author, year	Publication type	Country	Study aim(s)	Setting	Study design	Time-frame	Data sources	Population	N	Early psychosis definition	Forensic concept definition
			cooperation with treatment, and follow-up								
Meehan 2006 [46]	Journal article	England and Wales	Describe social and clinical characteristics, mental state features, offense details, and outcome in court of a national sample of people with schizophrenia convicted of homicide	Population-based (all homicides at the national level)	Cross-sectional	1996-1999	Government records (national homicide statistics), forensic psychiatric reports, police records, psychiatric hospital and community trust records, psychiatrist questionnaire	Persons convicted of homicide in England and Wales. The schizophrenia group was identified by a primary diagnosis of schizophrenia or other delusional disorder made by the psychiatric report writer or survey from mental health services.	1,594	No prior contact with mental health services for psychosis	Forensic psychiatric assessment
Millaud 1989 [47]	Journal article	Canada	Examine a population of patients with psychosis hospitalized in maximum security and who committed or attempted to commit murder to find the clinical elements associated with increased homicidal risk in the short term (one month)	Not reported	Case series	Not reported	Medico-legal files	People hospitalized in maximum security for committing homicide or attempting to commit homicide, meeting criteria for psychotic disorder	24	No prior contact with mental health services for psychosis	Admitted to forensic hospital or unit
Nielssen 2007 [48]	Journal article	Australia	Review homicides committed during psychotic illness in New South Wales over 10 years	Population-based covering state of New South Wales	Cross-sectional	1993-2002	Psychiatric reports from court proceedings	People who killed during psychotic episodes and were either found not guilty by reason of mental illness or who had the defense of mental illness available	88	No prior psychiatric treatment for psychosis	Found not guilty due to mental illness; Forensic psychiatric assessment
Nielssen 2009 [49]	Journal article	Australia	Examine the circumstances of homicides of children in New South Wales	Population-based, covering all child homicides in New South Wales	Cross-sectional	1991-2005	Crime statistics, court judgments, medicolegal reports, news reports	Homicides of people under the age of 18 between January 1, 1991, to December 31 2005	157	No prior psychiatric treatment for psychosis	Forensic psychiatric assessment
Nielssen 2010 [50]	Journal article	International	Estimate the rates of homicide during the first	Population-based	Systematic review;	1960-2008	Existing literature	Studies that reported a consecutive series of	849	No prior psychiatric	Found not guilty due to

Author, year	Publication type	Country	Study aim(s)	Setting	Study design	Time-frame	Data sources	Population	N	Early psychosis definition	Forensic concept definition
			episode of psychosis and after treatment		Meta-analysis			people who had committed a homicide/attempted homicide and were diagnosed with a psychotic illness were included if the study reported sufficient information to establish how many homicide offenders had received treatment before the homicide offense. Studies that reported all the homicides by those with a psychotic illness in a defined population were included.		treatment for psychosis; No prior contact with mental health services for psychosis; No prior hospitalization for psychosis; Other: Clinical definition (period after onset of psychosis and before remission from acute symptoms following a period of adequate treatment with antipsychotic medication)	mental illness; Admitted to forensic hospital or unit; Forensic psychiatric assessment
Nielssen 2011 [51]	Journal article	International (Australia, Canada, Finland, Netherlands)	Estimate the proportion of homicides committed during psychotic illness in which the victim was a stranger and the incidence of stranger homicide; examine homicide offenders with psychosis who had killed strangers, with reference to the proportion of those offenders who had previously received treatment with antipsychotic medication; compare characteristics of stranger homicide offenders with psychosis	Population-based	Case-control; Systematic review; Meta-analysis	1960-2008	Published studies	Studies which included all the homicides during psychotic illness in a defined population over a specified period and which also reported the number of subjects who killed strangers	84	No prior psychiatric treatment for psychosis; No prior contact with mental health services for psychosis; No prior hospitalization for psychosis	Forensic psychiatric assessment

Author, year	Publication type	Country	Study aim(s)	Setting	Study design	Time-frame	Data sources	Population	N	Early psychosis definition	Forensic concept definition
			with a control group who killed a family member								
Nielssen 2011 [52]	Journal article	Australia	Compare the characteristics (demographic, clinical, criminological) of two groups found not guilty on the grounds of mental illness: first episode psychosis versus those who previously received treatment	Population-based (state of New South Wales)	Cross-sectional	1992-2008	Court documents, including statements from police/witnesses, written judgments, psychiatric reports tendered in courts	Offenders with psychosis found NGMI for charges arising from assaults resulting in serious injury and homicides	272	No prior psychiatric treatment for psychosis	Found not guilty due to mental illness
Nielssen 2012 [53]	Journal article	Australia	Examine the association between mania and severe violence in a series of patients found not guilty by reason of mental illness	Population-based (state of New South Wales)	Cross-sectional	1992-2008	Court records for people found NGMI	Files of all those found NGMI of murder, attempted murder, wounding, or an assault resulting in serious injury in New South Wales between 1992 and 2008	272	No prior psychiatric treatment for psychosis	Found not guilty due to mental illness
Nielssen 2012 [54]	Journal article	International	Review the evidence for an association between first episode of psychosis and violence	Not reported	Narrative review	1970-2010	Existing literature	Published studies in English describing an association between violence and first-episode psychosis	N/A	No prior psychiatric treatment for psychosis	Found not guilty due to mental illness
Nielssen 2022 [55]	Journal article	Australia	Examine rates of homicide by people who were either convicted or found not guilty on the grounds of mental illness of homicide in New South Wales and examine the clinical characteristics of offenders	Population-based	Cross-sectional	1993-2016	Court records, national homicide statistics	Mentally ill offenders: All people found NGMI for homicide by the higher courts in New South Wales; non-mentally ill offenders: total number of offenders dealt with by the courts each year reported by the National Homicide Monitoring Program, minus the number of NGMI offenders.	2,159	No prior psychiatric treatment for psychosis	Found not guilty due to mental illness
Nijman 2003 [56]	Journal article	Germany and the Netherlands	Compare backgrounds of psychotic forensic patients with those of non-psychotic controls;	Two forensic hospitals	Cross-sectional	German sample: 2002; Dutch	Hospital patient files	The German sample consisted of all patients residing in the forensic psychiatric hospital on 1	308	No prior psychiatric treatment for psychosis	Admitted to forensic hospital or unit

Author, year	Publication type	Country	Study aim(s)	Setting	Study design	Time-frame	Data sources	Population	N	Early psychosis definition	Forensic concept definition
			explore whether psychotic patients committing the most severe offences have specific psychiatric and criminal antecedents distinguishing them from other psychotic offenders who did not cause death			sample: opening day onwards (1 November 1996)		January 2002. The Dutch sample consisted of all consecutive admissions to the forensic psychiatric hospital De Kijvelanden from its opening day onwards (1 November 1996).			
Nordström 2006 [57]	Journal article	Sweden	Describe homicides by people with schizophrenia to identify possible triggering factors	National, population-based	Cross-sectional	1992-2000	Forensic psychiatric evaluation reports and public court conviction protocols	Male homicide offenders (murder and manslaughter) undergoing pre-trial forensic psychiatric evaluation with diagnosis of schizophrenia between 1992 and 2000	48	No prior contact with mental health services for psychosis	Forensic psychiatric assessment
Penney 2023 [58]	Journal article	Canada	Examine mental health service utilization by persons who committed homicide and entered forensic care	Three hospitals in Ontario	Cross-sectional	2011-2021	Psychiatric reports, Ontario Review Board disposition documentation	All forensic patients adjudicated Not Criminally Responsible for an index offence of (or including) homicide and who were active inpatients or outpatients between 2011 and 2021	112	No prior contact with mental health services for psychosis	Found not guilty due to mental illness
Petroni 2022 [59]	Journal article	Italy	Present a case highlighting the psychopathological and contextual issues involved in the perpetration of matricide by a patient suffering from schizophrenia	Not reported	Case study	Not reported	Forensic pathology report, autopsy report, forensic psychiatric examination report	A case of matricide by a patient with a recent diagnosis of psychotic disorder	1	No prior hospitalization for psychosis	Found not guilty due to mental illness
Provoost 2022 [60]	Journal article	France	Explore the acting-out of homicidal mentally ill patients, with a particular focus on the characteristics of their delusions; assess the evolution of delusions	One forensic hospital	Cross-sectional	2015-2019, 1910-1914	Medical records, police reports, written, expert opinions, court transcripts, autopsy reports	Male, suffering from mental illness with delusions, committed homicide (or attempted) while experiencing delusions, hospitalized in the Henri Colin secure unit between 1910 and	40	No prior psychiatric treatment for psychosis	Admitted to forensic hospital or unit

Author, year	Publication type	Country	Study aim(s)	Setting	Study design	Time-frame	Data sources	Population	N	Early psychosis definition	Forensic concept definition
			across the 20th and early 21st centuries					1914, or between 2015 and 2019			
Purcell 2012 [61]	Journal article	Australia	Describe a service model for a pilot forensic satellite clinic embedded within an early intervention service	One outpatient/inpatient early intervention clinic in Western Melbourne (catchment covering population of 1 million)	Cross-sectional	2009-2010	Medical records	Patients referred by early intervention clinicians to the pilot forensic satellite clinic over the 12-month period and offered primary or secondary consultation	45	No prior psychiatric treatment for psychosis	Forensic patient; Contact with forensicare clinicians
Simpson 2006 [62]	Journal article	New Zealand	Examine the clinical and offence profiles of populations of homicide offenders with mental illness defined by the clinical and legal definitions of mentally abnormal homicide	Population-based (national)	Cross-sectional	1988-2000	Secondary government databases (police and law enforcement database, government, parole, Coroner's files, health administrative data)	Homicide offenders (non-mentally abnormal) and mentally abnormal homicides in New Zealand from 1988 to 2000 meeting either the clinical or legal definition. Legal definition: perpetrator received a mental health disposition from the courts and convicted for the offence of homicide. Clinical definition: perpetrators of homicide who had a prior or subsequent lifetime hospitalization for severe mental illness.	844	No prior hospitalization for psychosis	Found not guilty due to mental illness; Found unfit to stand trial
Singh 2024 [63]	Journal article	International	Describe violence in forensic psychiatric patients with psychosis, the factors associated with violence, and the relevant implications	Multiple	Systematic review	1999-2023	Published studies	Quantitative study designs with a sample consisting of forensic psychiatric patients above the age of 18 with findings of not criminally responsible or permanently unfit to stand trial or equivalent. Some fraction of the study participants needed to have	29 studies	Other: First psychotic episode	Forensic patient; Found not guilty due to mental illness; Found unfit to stand trial

Author, year	Publication type	Country	Study aim(s)	Setting	Study design	Time-frame	Data sources	Population	N	Early psychosis definition	Forensic concept definition
								psychotic disorder or psychotic symptoms, with stratified data available. Studies also needed to include violence as an outcome/focus of the study.			
Smedley 2010 [64]	Book chapter	United Kingdom	Present a case vignette showing the use of a psychological intervention in early psychosis in a secure setting	N/A	Other: Case vignette	N/A	N/A	N/A	N/A	No prior psychiatric treatment for psychosis	Admitted to forensic hospital or unit
Smith 1999 [65]	Journal article	United Kingdom	Examine the relationship between psychotic symptoms and sex offending in men with schizophrenia	Population-based (covering England and Wales)	Cross-sectional	May 1997	Case files from Mental Health Unit of the Home Office (court and police records, psychiatric records)	Inpatients residing in hospitals in England and Wales during May 1997 under legal category of mental illness following conviction for an index sex offence. Patients with a diagnosis of schizophrenia (without mental impairment or organic brain disease) were included. Exclusions: patients with other diagnoses, insufficient data, or diagnosed with first onset schizophrenia after their offence with no evidence of psychosis at time of offence.	80	No prior contact with mental health services for psychosis	Admitted to forensic hospital or unit
Tengström 2001 [66]	Journal article	Sweden	Identify characteristics that might be useful in designing treatment programs and management strategies for preventing further criminality and violence;	Population-based, covering Sweden	Cross-sectional	1988-1995	Reports of pretrial psychiatric assessments, National Police Register, Hospital	All men in Sweden who underwent a pretrial psychiatric assessment for the first time between 1988 and 1995 when accused of a violent offense, diagnosed with	272	No prior hospitalization for psychosis	Forensic psychiatric assessment

Author, year	Publication type	Country	Study aim(s)	Setting	Study design	Time-frame	Data sources	Population	N	Early psychosis definition	Forensic concept definition
			describe childhood and adolescent characteristics that distinguish early- and late-start offenders and identify possible targets of early intervention				Discharge Register, National Causes of Death Register, court documents	schizophrenia, and subsequently convicted of the offense			
Valença 2008 [67]	Journal article	Brazil	Evaluate a population of women diagnosed with primary psychotic disorders, hospitalized for homicide, or attempted homicide	One forensic hospital	Case series	July-December 2007	Patient interview, medical records, expert records, and opinions	All female patients diagnosed with a primary psychotic disorder who were serving a security measure for homicide or attempted homicide	8	No prior psychiatric treatment for psychosis	Admitted to forensic hospital or unit; Forensic psychiatric assessment
Valença 2011 [68]	Journal article	Brazil	Describe the cases of two women who faced criminal charges related to child homicide	Not reported	Case study	1987, 2006	Case records and clinical examination	Not reported	2	No prior psychiatric treatment for psychosis	Found not guilty due to mental illness; Admitted to forensic hospital or unit
Valença 2021 [69]	Journal article	Brazil	Describe cases of parricide, matricide, and filicide associated with presence of major mental disorders	Not reported	Case study	Not reported	Not reported	Brazilian cases of parricide, matricide, and filicide associated with the presence of major mental disorders and personality disorders	3	No prior psychiatric treatment for psychosis	Forensic psychiatric assessment
Valevski 1999 [70]	Journal article	Israel	Characterize patients hospitalized for homicide with a diagnosis of schizophrenia	Population-based (one high-security hospital covering whole NGMI for homicide with diagnosis of schizophrenia population in Israel)	Cross-sectional; Case-control	Not reported	Hospital records	Homicide group: inpatients at Gan Meged Hospital with schizophrenia found not guilty of homicide by reason of insanity. Control group: consecutive, non-homicidal inpatients with schizophrenia matched for age, sex, and duration of disease.	61	No prior contact with mental health services for psychosis	Found not guilty due to mental illness; Admitted to forensic hospital or unit

Author, year	Publication type	Country	Study aim(s)	Setting	Study design	Time-frame	Data sources	Population	N	Early psychosis definition	Forensic concept definition
Wang 2019 [71]	Journal article	China	Understand the demographic, clinical and criminological characteristics of Chinese homicide offenders with schizophrenia from a gender-based perspective	Population-based (covering all institutions in Hunan province)	Cross-sectional	2010-2016	Forensic records (medical and criminal files)	Aged 14 and above, diagnosis of schizophrenia, received forensic psychiatric assessment between Jan 1, 2010, and Dec 31, 2016. Exclusion criteria: schizophrenia developed after the crime, missing data, non-Hunan residents.	2,114	No prior psychiatric treatment for psychosis	Forensic psychiatric assessment
Wasser 2017 [72]	Other: Column	United States	Review evidence for heightened risk of aggression and criminal justice involvement in first episode psychosis and highlight opportunities for collaboration between mental health services and diversionary programs using a sequential intercept model	N/A	Narrative review; Other: Column	N/A	N/A	N/A	N/A	Other: First-episode psychosis	Forensic psychiatric assessment
Weithmann 2008 [73]	Journal article	Germany	Investigate the possibility of preventing offences in general inpatient psychiatric services	Two forensic hospitals covering region of Baden-Wurttemberg, Germany	Cross-sectional	2003	Medical and legal records	Male inpatients receiving treatment according to Section 63 of the Criminal Code with a schizophrenia spectrum disorder	75	No prior psychiatric treatment for psychosis	Admitted to forensic hospital or unit
Yee 2011 [74]	Journal article	Australia	Determine the proportion of severe violent assaults resulting in serious injury but not death that were committed by people with a psychotic illness; describe the characteristics of people with psychosis who commit severe	Population-based (state of New South Wales)	Cross-sectional	2006-2007	Court documents	Offences in court records between 2006 and 2007 in which there was a conviction or finding of NGMI for violence (attempted murder, malicious wounding or inflicting grievous bodily harm with intent, recklessly inflict grievous bodily harm or wounding,	661	No prior psychiatric treatment for psychosis; Other: patients in their first weeks of antipsychotic treatment who have yet to respond to	Found not guilty due to mental illness

Author, year	Publication type	Country	Study aim(s)	Setting	Study design	Time-frame	Data sources	Population	N	Early psychosis definition	Forensic concept definition
			violent offences; estimate the proportion of those with psychosis who commit serious violence who could be first episode psychosis					attempt to choke, assault causing grievous bodily harm, assault occasioning actual bodily harm).		treatment (onset of positive symptoms up to adequate period of treatment)	

NGMI = not guilty on the grounds of mental illness; DUP = duration of untreated psychosis; DSM-5; SSD, ICD-9; ICD-10

Table S4. Findings from each included evidence source related to forensic mental health service use and early psychosis (N = 74).

Author, year	Findings on forensic mental health service use and early psychosis
Ali 2024 [1]	A case of filicide was associated with psychosis onset during the peripartum period along with persecutory delusions, that was left untreated.
Alish 2007 [2]	There was a higher proportion of early psychosis in the schizophrenia-sex offences group relative to the schizophrenia non-sex-offence group (27.8% vs 12.5%)
Amani 2022 [3]	18% (n=11) of the sample (people with schizophrenia hospitalized for committing homicide) were early psychosis
Azoulay 2020 [4]	18/19 (95%) homicide offenders hospitalized without prior psychiatric treatment were early psychosis. Characteristics of patients whose first psychiatric contact is in the context of homicide: largely men, young adults, single, unemployed, and abuse substances. Duration of untreated psychosis was long and progressed for several months or even years.
Balcioglu 2024 [5]	In a sample of homicide offenders with schizophrenia spectrum disorder from Turkiye and Italy, the group from Italy had a significantly higher proportion of early psychosis cases compared to Turkiye (49% vs 12%).
Barbieri 2022 [6]	People suffering from Delusional Misidentification Syndromes (eg, "Doppelganger phenomenon") constitute a sub-category of psychiatric patients who may become violent towards others.
Bègue 2020 [7]	24% of offenders with schizophrenia undergoing psychiatric assessment were early psychosis. There were no differences in the proportion of early psychosis in the comorbid substance use disorder group vs no substance use disorder (27% vs 21%).
Belli 2010 [8]	7/49 (14.3%) homicide offenders with schizophrenia in a forensic hospital were early psychosis.
Böker & Häfner 1982 [9]	Among homicide offenders with schizophrenia in Germany over a 10-year period, 41% were early psychosis (116/284). Among homicide offenders diagnosed with affective psychoses, 51% (19/37) were early psychosis.
Bourget 2004 [10]	All cases presented with insidious illness onset, which went unrecognized until the homicidal event. Most patients (68%) were diagnosed with schizophrenia. Only 4 patients (18%) had past contacts with psychiatric services (for somatic complaints, substance abuse, and anxiety/depression). Only one patient had a history of violent behaviour.
Bourget 2004 [11]	All 4 cases were men who committed severe violence in early psychosis and experienced delusions of misidentification (Capgras Syndrome) which contributed to the violence towards others. Social withdrawal/self-isolation, and sexual preoccupation may also play a role.
Boylu 2025 [12]	In a sample of patients with delusional disorder undergoing forensic psychiatric assessment, 60% (89/150) were early psychosis. There was no significant difference in the proportion of people with early psychosis in the group with crime history vs no crime history (57% vs 60%, respectively).
Bruwer 2017 [13]	Among offenders hospitalized for a psychiatric assessment following domestic homicide and experiencing psychosis, 60% were early psychosis (6/10 experiencing psychosis at time of event; 6/21 in the whole sample of people with severe mental illness referred for psychiatric assessment following domestic homicide [29%]).
Carabellese 2014 [14]	1 of the 2 cases presented committed matricide in early psychosis. The Capgras' delusion experienced by both cases contributed to the matricide.
Carabellese 2021 [15]	23.1% of the schizophrenia spectrum disorder group of homicide/attempted homicide offenders were early psychosis. Among all forensic psychiatric assessments for homicide/attempted homicide, early psychosis makes up a small proportion (9/187, 4.8%)

Author, year	Findings on forensic mental health service use and early psychosis
Chen 2018 [16]	42/107 (39%) of people with schizophrenia who committed homicide and referred for psychiatric assessment had never received any mental health treatment (i.e., were early psychosis).
Davies 2006 [17]	Powerlessness and lack of control of the situation may be risk factors for the genesis of violence in people with early psychosis
Dolan 1996 [18]	Among homicide offenders in a forensic hospital with psychotic disorder, 22% (12/54) were early psychosis. Within the full forensic sample (psychotic and non-psychotic homicide offenders), early psychosis accounted for 10% of the sample (12/120).
Erb 2001 [19]	Across both time periods, 40% (125/313) of people with schizophrenia committing or attempting to commit homicide were early psychosis. Within the older cohort, 116/284 (41%) were early psychosis, and within the more recent cohort 9/29 (31%) were early psychosis. Data from the recent period suggest lack of appropriate services for chronic high-risk patients and the non-use of mental health services in the early psychosis patients were associated with homicide.
Golenkov 2011 [20]	In Chuvashia about 1 in 200 patients with early schizophrenia present for treatment after committing homicide (34/6700 estimated in the region). The rate of previously treated homicide was estimated at 1 in 1259 (rate of homicide in early schizophrenia is 6 times higher than in previously treated patients).
Goodhand 2024 [21]	Among those found not guilty due to mental illness with a schizophrenia-related diagnosis, 23% were early psychosis. Although the majority of the sample had a psychotic disorder, it was not diagnosed in the majority of prior emergency department visits (58%) and outpatient visits (67%), and in 31% of hospitalizations prior to the offence, suggesting that emerging or underlying psychosis was often missed or attributed to other conditions.
Gralton 2000 [22]	Schizophrenia may go unrecognized in forensic samples of people with mild and borderline learning disability, and may explain violent offending behaviour.
Grungberg 1978 [23]	3/9 (33%) psychotic homicides were early psychosis.
Hadidy 2012 [24]	Among homicide offenders with schizophrenia undergoing psychiatric assessment, 27% were early psychosis. There were no differences in the proportion of early psychosis cases between the homicide group and non-homicide group with schizophrenia (27.3% vs 29.7%).
Hardwick 2003 [25]	The patients whose first ever psychiatric admission was to medium security accounted for 5% of the total number of admissions over the total study period. Among those who were in their first episode of psychiatric illness, 75%-85% were early psychosis (depending on time period).
He 2022 [26]	87 (26%) of the patients with schizophrenia undergoing forensic psychiatric assessment due to interpersonal violence were early psychosis (no previous treatment definition), and for 45% of the sample it was a first hospitalization.
Hodgins 2004 [27]	Among men with schizophrenia spectrum disorders admitted to a forensic hospital, 22% (35/158) were early psychosis at the time of admission (i.e., had no prior admission in general psychiatry). The group of forensic patients that had prior admissions did not differ significantly from the early psychosis group with respect to age and proportion born outside the site country. Those treated only in forensic psychiatric care committed 2.7 violent and 8.1 non-violent offences that gained them admission to a forensic hospital (the previously hospitalized group committed an average of 2.7 violent and 2.7 non-violent offences that gained them admission). 22% of the previously hospitalized group and 26% of the early psychosis group were admitted to forensic hospital following commission of murder or attempted murder.
Humphreys 1994 [28]	15/31 patients (48%) committed offences associated with psychotic symptoms or were ill at the time of the offence. Only 6/15 patients (40%) were diverted to forensic mental health services via hospital order. In other cases, the mental disorder went unnoticed.

Author, year	Findings on forensic mental health service use and early psychosis
Jacobshagen 2024 [29]	29% of female offenders in a forensic hospital with schizophrenia spectrum disorder were early psychosis (no prior antipsychotic treatment). This proportion was significantly higher than non-offender patients with schizophrenia spectrum disorder (none were early psychosis).
Kachouchi 2018 [30]	Among people with schizophrenia hospitalized for homicide, 6/30 (20%) were early psychosis. There was a significantly higher proportion of early psychosis patients in the homicide group compared to the non-forensic hospitalized sample.
Kirchebner 2023 [31]	26% of offender patients with schizophrenia spectrum disorder were early psychosis (inpatient definition), which was significantly more than the non-offender schizophrenia spectrum disorder group (5%).
Koks 2024 [32]	In a sample of forensic outpatients with no prior diagnosis of psychotic disorder and screened for psychosis symptoms, 9% were found to be early psychosis.
Laajasalo 2005 [33]	Among homicide offenders with schizophrenia in Finland, 16% (17/109) were early psychosis.
Large 2008 [34]	The proportion of early psychosis homicides ranged from 13% to 76%. Longer average duration of untreated psychosis at the country level was associated with a higher proportion of patients who committed homicide prior to treatment.
LeBihan 2004 [35]	38% of men hospitalized to a maximum-security hospital due to parricide were early psychosis.
Leong 1995 [36]	12/25 (48%) homicide offenders with psychosis in Los Angeles County were early psychosis cases
Levine 2008 [37]	5.6% of patients were admitted with forensic status at first admission (8.1% of males and 1.8% of females).<br>Over 4- to 18-year follow up: 35% of males and 16% of females with an index forensic admission had a second forensic admission. A forensic index admission was associated with a higher average number of hospital days per year and longer length of first stay relative to voluntary and involuntary index admissions. For males, a forensic index admission was associated with more admissions per illness year.
Lewis 2003 [38]	Among women with psychosis committing filicide and undergoing psychiatric assessment, 14% were early psychosis. Among all women undergoing psychiatric assessment for filicide in the state of Michigan, 9% were early psychosis.
Liem 2012 [39]	Among people with psychotic disorder who committed homicide in the Netherlands, 34/61 (56%) were early psychosis.
Machetanz 2023 [40]	40% of offender patients with schizophrenia spectrum disorder were early psychosis (no prior treatment definition), which was significantly higher than non-offender patients with schizophrenia spectrum disorder (6.5%).
Macmillan 1987 [41]	4.7% were hospitalized under a forensic section of the Mental Health Act. Among patients with police contact prior to admission, 22% were hospitalized under forensic section of the Mental Health Act.
Markopoulou 2021 [42]	21/78 (27%) of patients found not guilty by reason of insanity with a psychotic disorder were early psychosis. Early psychosis patients were younger, more likely to have recently experienced a stressful life event, and more likely to have assaulted a member of the family. Patients with higher hostility scores in the aggression questionnaire remained at risk for committing a crime during the course of illness.
Markopoulou 2023 [43]	Among offenders found not guilty by reason of insanity hospitalized in northern Greece, committing the offence in the first episode of psychosis was not associated with a history of conduct disorder (30% were early psychosis in the conduct disorder group vs 25% in the no conduct disorder group).
Markopoulou 2024 [44]	Among offenders found not guilty by reason of insanity hospitalized in northern Greece, committing the offence in the first episode of psychosis was not associated with a history of conduct disorder (30% were early psychosis in the conduct disorder group vs 25% in the no conduct disorder group).

Author, year	Findings on forensic mental health service use and early psychosis
McGovern 1991 [45]	On first psychiatric admission, Black-Caribbeans were more likely to be detained under a forensic section compared to the white group (10% vs 0%) and over 4- to 8- year follow-up. 21% of Black Caribbeans and 3% of the white group were admitted under forensic sections at any point over the study period. Only males were detained in either group, with 1/3 of Black-Caribbean males having been detained (and 5% of the white group).
Meehan 2006 [46]	Among homicide offenders with schizophrenia, 28% were early psychosis patients (proportion is higher if including the 12 cases with prior contact but schizophrenia not recognized: 36/85 [42%]). Among all homicide offenders, early psychosis accounted for a very small proportion (1.5%; 24/1594). In the month before the offense, those with no previous service contact were more likely to have experienced an increase in the conviction with which their delusions were held compared to the previously treated group (52% vs 25%).
Millaud 1989 [47]	Among homicide offenders with schizophrenia, 28% were early psychosis patients (proportion is higher if including the 12 cases with prior contact but schizophrenia not recognized: 36/85 [42%]). Among all homicide offenders, early psychosis accounted for a very small proportion (1.5%; 24/1594). In the month before the offense, those with no previous service contact were more likely to have experienced an increase in the conviction with which their delusions were held compared to the previously treated group (52% vs 25%).
Nielssen 2007 [48]	People in first episodes of mental illness are at greater risk of committing serious violence than those in subsequent episodes (54 people (61%) of homicide offenders with psychotic illness were in their first episode of psychosis and 61 (69%) were in their first year of illness).
Nielssen 2009 [49]	26/151 (17.2%) of offenders committed child homicide during a psychotic illness. Early psychosis patients were over-represented (58%, 15/26). 86% of early psychosis offenders had contact with some form of health service in the 2 weeks before the homicide, and 33% had contact with a mental health service at some point in the past. There is a higher proportion of women in the early psychosis group vs previously treated (93% vs 46%).
Nielssen 2010 [50]	Approximately 4 in 10 of the homicides committed by people with a psychotic illness occur in early psychosis (i.e., before treatment; pooled estimate: 38.5%, 95% CI 31.1%-46.5%). Approximately 1 in 700 people with psychosis commit a homicide in early psychosis, and approximately 1 in 10 000 patients with psychosis who have received treatment will commit a homicide each year; the rate of homicide in psychosis before treatment is approximately 15 times higher than the annual rate after treatment.
Nielssen 2011 [51]	Within both the stranger homicide group and the family homicide group, there was a large proportion of early psychosis (64% vs 52%; no significant difference). Never-treated homicide offenders were more likely to hold delusional beliefs about the victim compared to the family homicide offenders, but there were no other differences found in demographic, illness, or offence characteristics.
Nielssen 2011 [52]	Among people found NGMI of serious violent offence with a psychotic disorder, 46% were early psychosis. There was a higher proportion of early psychosis homicide offenders (52%) than among offenders who committed severe nonlethal assaults (39%), suggesting an association between severe violence and early psychosis. Early psychosis NGMI patients were younger, more likely to come from non-English speaking countries and to have used a firearm in the offense compared to previously treated NGMI psychosis patients.
Nielssen 2012 [53]	4.4% (12/272) people found NGMI of severe violent offence were in a manic state at the time of the offence; 4/12 (33.3%) manic offenders were early psychosis.
Nielssen 2012 [54]	About one-half of psychotic patients who commit the most severe forms of violent offences in association with psychosis are early psychosis, suggesting an increased risk of serious violence in this population.
Nielssen 2022 [55]	About one quarter of NGMI homicide offenders with severe mental illness were early psychosis; 4 in 10 NGMI offenders had no record of having been treated with antipsychotic medication prior to the offence.

Author, year	Findings on forensic mental health service use and early psychosis
Nijman 2003 [56]	8.1% (25/308) of forensic patients were early psychosis at the time of the index offence. Among patients with psychotic illness, 22.5% (25/111) were early psychosis. Comparing lethal psychotic offenders with non-lethal offenders, 15% were early psychosis compared to 26% in the non-lethal group (not statistically significant).
Nordström 2006 [57]	21% of homicide offenders with schizophrenia undergoing forensic psychiatric assessment were early psychosis
Penney 2023 [58]	Among homicide offenders with psychosis found not guilty by reason of insanity in Ontario, 24.3% were early psychosis; this group tended to be younger at the time of the homicide, have a shorter duration of illness, and were less likely to have prior arrests or criminal charges.
Petroni 2022 [59]	Persecutory delusions with contents of transformation/misidentification involving the mother contributed to matricide in an early psychosis case.
Provoost 2022 [60]	Within the historical sample of men hospitalized for homicide, the prevalence of early psychosis was high (70%). Within the modern sample, the prevalence of early psychosis was lower (47%).
Purcell 2012 [61]	In a sample of youth referred to a pilot forensic clinic embedded within early intervention services for mental illness, 40% were early psychosis.
Simpson 2006 [62]	Among psychotic homicide offenders in New Zealand, just under one-third (28%) were early psychosis (first admitted at the time of the offence). This accounts for 9.6% (18/187) of all mentally abnormal homicides, and 2.1% of all homicides, (18/844).
Singh 2024 [63]	Early psychosis offenders were more likely to commit severe index offenses, driven by delusions and persecutory ideation, often preceded by a stressful life event and followed by a suicide attempt. They were also more likely to be born overseas in a non-English-speaking country, report depressed mood at the time of the offense, and use a firearm. In contrast, later offenders had more chronic psychopathology, worse outcomes, and were more likely to have prior convictions.
Smedley 2010 [64]	The case vignette provides an example for clinicians, illustrating the use of psychological intervention with a young person experiencing early psychosis in a secure hospital setting.
Smith 1999 [65]	Among men with schizophrenia hospitalized for sex offences 8/80 (10%) were early psychosis. There was a higher proportion of early psychosis patients in the white group vs Black-Caribbean, using criteria of whether schizophrenia was ever diagnosed (29% vs 5%; there was also a higher proportion using past psychiatric contact criteria [84% vs 95%] but this was not statistically significant).
Tengström 2001 [66]	In a sample of male offenders with schizophrenia convicted of violent offense, 22% were early psychosis (61/272). There were no differences in the prevalence of early psychosis between the early and late start offender groups with schizophrenia (23% vs 22%).
Valença 2008 [67]	In a case series of women hospitalized for homicide/attempted homicide in Brazil, 2/8 (25%) were early psychosis at the time of the offence.
Valença 2011 [68]	In a case of filicide occurring in the context of early psychosis, the patient had limited social support, living in a situation with family conflict, presented persecutory delusions and auditory hallucinations. The early psychosis case was different from the chronic schizophrenia case in that there was no history of violent behaviour or substance/alcohol abuse.
Valença 2021 [69]	Case study of parricide occurring in the context of early psychosis, illustrating that most crimes committed by individuals with schizophrenia occur in the family setting and in the initial phase of the disease. Paranoid ideation played a role in the violent behavior, as the case displayed persecutory delusions in relation to neighbors and the police. The case also illustrates the "late starter" trajectory to violent behaviour in schizophrenia (patients without a prior history of violence who present this behaviour at the beginning of illness, where positive symptoms explain the violence).

**Author, year**	**Findings on forensic mental health service use and early psychosis**
Valevski 1999 [70]	Among patients with schizophrenia found NGMI for homicide, 13/33 (39%) were early psychosis.
Wang 2019 [71]	Within the sample of patients with schizophrenia undergoing forensic psychiatric examination, 28% were early psychosis. Proportions were similar in within homicide and non-homicide offenders (28% in both groups). Within homicide offenders, stratified by sex, there were no significant differences in the proportion of the sample in early psychosis in males vs females (28% vs 29%). There were also no significant differences in the proportion of early psychosis cases among male offenders, when comparing homicide vs non-homicide groups (28% vs 29%). The same finding held within female offenders (26% early psychosis in homicide group vs 25% in non-homicide group).
Wasser 2017 [72]	Forensic evaluations are an intercept point along the sequential intercept model in which to divert people experiencing early psychosis from incarceration.
Weithmann 2008 [73]	Among forensic inpatients in Germany with a schizophrenia spectrum disorder, a small proportion were early psychosis (5.3%)
Yee 2011 [74]	Of the severe violent offenders with a diagnosis of psychotic illness, 22% (16/74) were early psychosis cases, comprising 2.4% of all severe non-lethal assaults. Compared to psychosis patients who previously received treatment, the early psychosis group were more likely to be female (13% vs 3%), from non-English speaking country (31% vs 10%), less likely to have previous criminal conviction (63% vs 90%), and older at time of psychosis onset (27 vs 23 years old).

NGMI = not guilty on the grounds of mental illness

References

1. Ali O., Islam S., Raai H., Troneci L. (2024) Postpartum Psychosis and Maternal Filicide-Case Report and Literature Review. Eur Psychiatry 67:S810. https://doi.org/10.1192/j.eurpsy.2024.1688

2. Alish Y, Birger M, Manor N, et al (2007) Schizophrenia sex offenders: A clinical and epidemiological comparison study. Int J Law Psychiatry 30:459–466. https://doi.org/10.1016/j.ijlp.2007.09.006

3. Amani M, Boumeslout S, Bencharif M, Belkourissat M (2022) Characteristics and particularities of homicides committed by schizophrenics. Ann Méd Psychol 180:S66–S74. https://doi.org/10.1016/j.amp.2020.10.007

4. Azoulay M., Obadia S., Raymond S. (2020) Homicide as the inaugural symptom for psychiatric disorder: Truth or myth? Evol Psychiatr (Paris) 85:179–192. https://doi.org/10.1016/j.evopsy.2019.12.005

5. Balcioglu YH, Margari A, Yildiz A, et al (2024) Homicides by offenders with psychotic illness in Italy and Turkiye: A comparison of offender and crime-scene profiles. Int J Law Psychiatry 97:102041. https://doi.org/10.1016/j.ijlp.2024.102041

6. Barbieri C, Rocca G, Bosco C, et al (2022) The Doppelganger phenomenon and death: a peculiar case of homicide by a subject with first-episode psychosis. Forensic Sci Res 7:798–802. https://doi.org/10.1080/20961790.2022.2055827

7. Bègue C, Bouthier M, Mahé V (2020) Rôle de l'usage de substances psycho-actives dans la commission d'infractions pénales par les sujets atteints de schizophrénie. Evol Psychiatr (Paris) 85:217–228. https://doi.org/10.1016/j.evopsy.2020.02.002

8. Belli H, Ozcetin A, Ertem U, et al (2010) Perpetrators of homicide with schizophrenia: sociodemographic characteristics and clinical factors in the eastern region of Turkey. Compr Psychiatry 51:135–141. https://doi.org/10.1016/j.comppsych.2009.03.006

9. Böker W (Wolfgang), Häfner H (Heinz) (1982) Crimes of violence by mentally abnormal offenders : a psychiatric and epidemiological study in the Federal German Republic. Cambridge University Press, Cambridge [Cambridgeshire] ;

10. Bourget D, Labelle A, Gagné P, Tessier P (2004) First-episode psychosis and homicide: a diagnostic challenge. Can Psychiatr Assoc Bull 36:6–9

11. Bourget D, Whitehurst L (2004) Capgras Syndrome: A Review of the Neurophysiological Correlates and Presenting Clinical Features in Cases Involving Physical Violence. Can J Psychiatry 49:719–725. https://doi.org/10.1177/070674370404901102

12. Boylu ME, Kavla Y, Şahin HÇ, Duran A (2025) Comparison of sociodemographic, clinical and forensic characteristics of delusional disorder cases with and without violent crime history: a comparative-descriptive retrospective study from Turkey. Psychiatry Psychol Law 1–15. https://doi.org/10.1080/13218719.2024.2421161

13. Bruwer M (2017) Characteristics of domestic homicide perpetrated by persons with severe mental illness-a forensic psychiatry observation population-based study. open.uct.ac.za

14. Carabellese F, Rocca G, Candelli C, Catanesi R (2014) Mental illness, violence and delusional misidentifications: The role of Capgras' syndrome in matricide. J Forensic Leg Med 21:9–13. https://doi.org/10.1016/j.jflm.2013.10.012

15. Carabellese F, Mandarelli G, Felthous AR, Catanesi R (2021) Forensic psychiatric evaluation of 187 homicidal assailants with and without a schizophrenia spectrum disorder: Clinical, criminological and behavioral characteristics. Behav Sci Law 39:190–204. https://doi.org/10.1002/bsl.2515

16. Chen X, Zhang X, Wong S, et al (2018) Characteristics of alleged homicide offenders with and without schizophrenia in Sichuan, China. Crim Behav Ment Health 28:202–215. https://doi.org/10.1002/cbm.2054

17. Davies G (2006) Powerlessness as a risk factor for violence in first episode schizophrenia: Two illustrative cases. Am J Forensic Psychiatry 27:29–35

18. Dolan M, Parry J (1996) A survey of male homicide cases resident in an English special hospital. Med Sci Law 36:249–258. https://doi.org/10.1177/002580249603600312

19. Erb M, Hodgins S, Freese R, et al (2001) Homicide and schizophrenia: maybe treatment does have a preventive effect. Crim Behav Ment Health 11:6–26. https://doi.org/10.1002/cbm.366

20. Golenkov A, Large M, Nielssen O, Tsymbalova A (2011) Characteristics of homicide offenders with Schizophrenia from the Russian Federation. Schizophr Res 133:232–237. https://doi.org/10.1016/j.schres.2011.07.008

21. Goodhand C, Lyons G, Johnson A, et al (2024) Patterns and correlates of health service contact prior to serious offences by people with severe mental illness. Aust N Z J Psychiatry 58:775–786. https://doi.org/10.1177/00048674241254213

22. Gralton E, James A, Crocombe J (2000) The diagnosis of schizophrenia in the borderline learning-disabled forensic population: six case-reports. J Forensic Psychiatry 11:185–197. https://doi.org/10.1080/095851800362463

23. Grungberg F, Klinger BI, Grumet BR (1978) Homicide and community-based psychiatry. J Nerv Ment Dis 166:868–874. https://doi.org/10.1097/00005053-197812000-00006

24. Hadidy MA el (2012) Schizophrenia with and without homicide: a clinical comparative study. J Forensic Psychiatry Psychol 23:95–107. https://doi.org/10.1080/14789949.2011.650183

25. Hardwick E., Gray D., Humphreys M. (2003) First ever admission to medium security. Med Sci Law 43:345–349

26. He Y, Gu Y, Yu M, et al (2022) Research on interpersonal violence in schizophrenia: based on different victim types. BMC Psychiatry 22:. https://doi.org/10.1186/s12888-022-03820-7

27. Hodgins S., Muller-Isberner R. (2004) Preventing crime by people with schizophrenic disorders: The role of psychiatric services. Br J Psychiatry 185:245–250. https://doi.org/10.1192/bjp.185.3.245

28. Humphreys M, Johnstone E, Macmillan F (1994) Offending among first episode schizophrenics. J Forensic Psychiatry 5:51–61. https://doi.org/10.1080/09585189408410897https://dx.doi.org/10.1080/09585189408410897

29. Jacobshagen L, Machetanz L, Kirchebner J (2024) Differences between criminal offender versus non-offender female patients with schizophrenia spectrum disorder: a retrospective cohort study. Arch Womens Ment Health. https://doi.org/10.1007/s00737-024-01477-7

30. Kachouchi A, Sebbani M, Salim S, et al (2018) [Risk factors related to homicide in Moroccan patients with schizophrenia]. L'Encephale 44:409–414. https://doi.org/10.1016/j.encep.2017.07.001

31. Kirchebner J, Lau S, Machetanz L (2023) Offenders and non-offenders with schizophrenia spectrum disorders: Do they really differ in known risk factors for aggression? Front Psychiatry 14:1145644. https://doi.org/10.3389/fpsyt.2023.1145644

32. Koks C, Rijckmans M, Dam AV (2024) Double Prevention in Outpatient Forensic Care; Early Psychotic Experiences and Its Relationship with Aggression and Antisocial Personality Disorder. Psychology 15:1296–1308. https://doi.org/10.4236/psych.2024.158077

33. Laajasalo T, Hakkanen H (2005) Offence and offender characteristics among two groups of Finnish homicide offenders with schizophrenia: Comparison of early- and late-start offenders. J Forensic Psychiatry Psychol 16:41–59. https://doi.org/10.1080/1478994042331327679

34. Large M, Nielssen O (2008) Evidence for a relationship between the duration of untreated psychosis and the proportion of psychotic homicides prior to treatment. Soc Psychiatry Psychiatr Epidemiol 43:37–44. https://doi.org/10.1007/s00127-007-0274-0

35. Le Bihan P, Bénézech M (2004) Degré d'organisation du crime de parricide pathologique : mode opératoire, profil criminologique. À propos de 42 observations. Ann Méd Psychol 162:615–625. https://doi.org/10.1016/j.amp.2004.06.014

36. Leong GB, Silva JA (1995) A psychiatric-legal analysis of psychotic criminal defendants charged with murder. J Forensic Sci 40:445–448

37. Levine SZ (2008) Population-based examination of the relationship between type of first admission for schizophrenia and outcomes. Psychiatr Serv 59:1470–1473

38. Lewis CF, Bunce SC (2003) Filicidal mothers and the impact of psychosis on maternal filicide. J Am Acad Psychiatry Law 31:459–470

39. Liem MCA, Vinkers DJ (2012) [Homicide committed by psychotic defendants in the Netherlands]. Tijdschr Voor Psychiatr 54:509–516

40. Machetanz L, Hofmann AB, Möhrke J, Kirchebner J (2023) Offenders and non-offenders with schizophrenia spectrum disorders: the crime-preventive potential of sufficient embedment in the mental healthcare and support system. Front Psychiatry 14:1231851. https://doi.org/10.3389/fpsyt.2023.1231851

41. Macmillan JF, Johnson AL (1987) Contact with the police in early schizophrenia: its nature, frequency and relevance to the outcome of treatment. Med Sci Law 27:191–200

42. Markopoulou M, Karakasi V, Garyfallos G, et al (2021) Research findings on Greek forensic patients found not guilty by reason of insanity. A juxtaposition of patients who committed a criminal offense during their first psychotic episode with those who did so later in the course of their illness. Int J Law Psychiatry 75:. https://doi.org/10.1016/j.ijlp.2021.101673

43. Markopoulou M, Chatzinikolaou F, Karakasi M-V, et al (2023) Psychosis and conduct disorder in Greek forensic patients found not guilty by reason of insanity: Differences between patients with and those without a history of conduct disorder in childhood or adolescence. Int J Law Psychiatry 86:101855. https://doi.org/10.1016/j.ijlp.2022.101855

44. Markopoulou M, Chatzinikolaou F, Avramidis A, et al (2024) Parricide and homicide NGRI offenders: How do they differ? Med Sci Law 1–8. https://doi.org/10.1177/00258024241286743

45. McGovern D, Cope R (1991) Second generation Afro-Caribbeans and young whites with a first admission diagnosis of schizophrenia. Soc Psychiatry Psychiatr Epidemiol 26:95–99. https://doi.org/10.1007/BF00791535

46. Meehan J, Flynn S, Hunt IM, et al (2006) Perpetrators of Homicide With Schizophrenia: A National Clinical Survey in England and Wales. Psychiatr Serv 57:1648–1651. https://doi.org/10.1176/ps.2006.57.11.1648

47. Millaud F (1989) [Homicide in the psychotic patient: a study of 24 cases with reference to prediction of short-term outcome]. Can J Psychiatry Rev Can Psychiatr 34:340–346. https://doi.org/10.1177/070674378903400415

48. Nielssen O, Westmore B, Large M, Hayes R (2007) Homicide during psychotic illness in New South Wales between 1993 and 2002. Med J Aust 186:301–304. https://doi.org/10.5694/j.1326-5377.2007.tb00905.x

49. Nielssen O, Large M, Westmore B, Lackersteen S (2009) Child homicide in New South Wales from 1991 to 2005. Med J Aust 190:7–11. https://doi.org/10.5694/j.1326-5377.2009.tb02252.x

50. Nielssen O, Large M (2010) Rates of homicide during the first episode of psychosis and after treatment: a systematic review and meta-analysis. Schizophr Bull 36:702–712. https://doi.org/10.1093/schbul/sbn144

51. Nielssen O, Bourget D, Laajasalo T, et al (2011) Homicide of Strangers by People with a Psychotic Illness. Schizophr Bull 37:572–579. https://doi.org/10.1093/schbul/sbp112

52. Nielssen OB, Yee NL, Millard MM, Large MM (2011) Comparison of first-episode and previously treated persons with psychosis found NGMI for a violent offense. Psychiatr Serv 62:759–764. https://doi.org/10.1176/ps.62.7.pss6207_0759

53. Nielssen O, Malhi G, Large M (2012) Mania, homicide and severe violence. Aust N Z J Psychiatry 46:357–363. https://doi.org/10.1177/0004867411433217

54. Nielssen OB, Malhi GS, McGorry PD, Large MM (2012) Overview of violence to self and others during the first episode of psychosis. J Clin Psychiatry 73:. https://doi.org/10.4088/JCP.11r07036

55. Nielssen O, Lyons G, Oldfield K, et al (2022) Rates of homicide and homicide associated with severe mental illness in NSW between 1993 and 2016. Aust N Z J Psychiatry 56:836–843. https://doi.org/10.1177/00048674211040016

56. Nijman H, Cima M, Merckelbach H (2003) Nature and antecedents of psychotic patients' crimes. J Forensic Psychiatry Psychol 14:542–553. https://doi.org/10.1080/1478994031000152754

57. Nordström A, Dahlgren L, Kullgren G (2006) Victim relations and factors triggering homicides committed by offenders with schizophrenia. J Forensic Psychiatry Psychol 17:192–203. https://doi.org/10.1080/14789940600631522

58. Penney SR, Lam AA, Kolla N, et al (2023) Homicide in the context of psychosis: analysis of prior service utilisation and age at onset of illness and violence. BJPsych Open 9:e171–e171. https://doi.org/10.1192/bjo.2023.567

59. Petroni G., Mandarelli G., Marasco M., et al (2022) From overkill to beheading: A case report of a schizophrenic matricide. J Forensic Sci 67:404–407. https://doi.org/10.1111/1556-4029.14829

60. Provoost E, Raymond S, Gasman I (2022) Homicides committed by delusional patients in the early 20th and 21st centuries: A study conducted in a French secure unit. J Forensic Sci 67:265–274. https://doi.org/10.1111/1556-4029.14892

61. Purcell R., Fraser R., Greenwood-Smith C., et al (2012) Managing risks of violence in a youth mental health service: A service model description. Early Interv Psychiatry 6:469–475. https://doi.org/10.1111/j.1751-7893.2012.00372.x

62. Simpson AIF, Skipworth J, Mckenna B, et al (2006) Mentally abnormal homicide in New Zealand as defined by legal and clinical criteria: A national study. Aust N Z J Psychiatry 40:804–809. https://doi.org/10.1111/j.1440-1614.2006.01887.x

63. Singh A, Pereira W, Birk S, et al (2024) Characterizing the relationship between psychosis and violence in the forensic psychiatric population: a systematic review. CNS Spectr 29:335–346. https://doi.org/10.1017/S1092852924000488

64. Smedley K (2010) Cognitive behaviour therapy with adolescents in secure settings. Psychol Ther Prisons Secure Settings 71–101

65. Smith AD, Taylor PJ (1999) Serious sex offending against women by men with schizophrenia: Relationship of illness and psychotic symptoms to offending. Br J Psychiatry 174:233–237. https://doi.org/10.1192/bjp.174.3.233

66. Tengström A, Hodgins S, Kullgren G (2001) Men with Schizophrenia Who Behave Violently: The Usefulness of an Early-Versus Late-Start Offender Typology. Schizophr Bull 27:205–218. https://doi.org/10.1093/oxfordjournals.schbul.a006867

67. Valença AM (2008) Aspectos psicopatológicos e forenses de mulheres homicidas com diagnóstico de transtornos psicóticos primários—Estudo de série de casos. [Psychopathologic and forensic features of female murderers with a diagnosis of primary psychotic disorders—A case series.]. J Bras Psiquiatr 57:253–260. https://doi.org/10.1590/S0047-20852008000400005

68. Valença AM, Mendlowicz MV, Nascimento I, Nardi AE (2011) Filicide, Attempted Filicide, and Psychotic Disorders. J Forensic Sci 56:551–554. https://doi.org/10.1111/j.1556-4029.2010.01645.x

69. Valença AM, Carvalho de Oliveira G, Telles LE de B, et al (2021) Matricide, parricide, and filicide: Are major mental disorders or personality disorders involved? Assessment of criminal responsibility in Brazilian cases. J Forensic Sci 66:2048–2053. https://doi.org/10.1111/1556-4029.14745

70. Valevski A, Averbuch I, Radwan M, et al (1999) Homicide by schizophrenic patients in Israel. Eur Psychiatry 14:89–92. https://doi.org/10.1016/S0924-9338(99)80723-8

71. Wang J, Zhang S, Zhong S, et al (2019) Gender differences among homicide offenders with schizophrenia in Hunan Province, China. Psychiatry Res 271:124–130. https://doi.org/10.1016/j.psychres.2018.11.039

72. Wasser T, Pollard J, Fisk D, Srihari V (2017) First-episode psychosis and the criminal justice system: Using a sequential intercept framework to highlight risks and opportunities. Psychiatr Serv 68:994–996. https://doi.org/10.1176/appi.ps.201700313

73. Weithmann G, Traub H-J (2008) Psychiatric antecedents of forensic patients with schizophrenia - Framework conditions of crime prevention by General Psychiatry. Forensische Psychiatr Psychol Kriminol 2:112–119. https://doi.org/10.1007/s11757-008-0071-y

74. Yee NYL, Large MM, Kemp RI, Nielssen OB (2011) Severe non-lethal violence during psychotic illness. Aust N Z J Psychiatry 45:466–472. https://doi.org/10.3109/00048674.2011.541417
